# Electric Scooter and Electric Bicycle Injuries in Children and Adolescents: A Narrative Review of Epidemiology, Injury Patterns, Clinical Outcomes, and Prevention

**DOI:** 10.3390/healthcare14152367

**Published:** 2026-08-03

**Authors:** Marko Bašković, Matej Lacković, Jana Buzuk, Bianka Dujić, Danijela Jurić, Kristina Jurković, Karla Pehar, Sara Vuković, Marta Borić Krakar

**Affiliations:** 1 School of Medicine, Catholic University of Croatia, Ilica 242, 10000 Zagreb, Croatia; 2 Department of Pediatric Surgery, Children’s Hospital Zagreb, Ulica Vjekoslava Klaića 16, 10000 Zagreb, Croatia; 3 School of Medicine, University of Zagreb, Šalata 3, 10000 Zagreb, Croatia; 4 Scientific Centre of Excellence for Reproductive and Regenerative Medicine, School of Medicine, University of Zagreb, Šalata 3, 10000 Zagreb, Croatia; 5 Croatian Academy of Medical Sciences, Kaptol 15, 10000 Zagreb, Croatia; 6 Referral Center for Pediatric Traumatology, Children’s Hospital Zagreb, Ulica Vjekoslava Klaića 16, 10000 Zagreb, Croatia; 7 Sector for Hospital Health Care, Ministry of Health, Ksaver 200a, 10000 Zagreb, Croatia

**Keywords:** electric scooter, e-scooter, electric bicycle, e-bike, micromobility, paediatric injury, adolescent, traumatic brain injury, helmet, injury prevention

## Abstract

Electric scooters (e-scooters) and electric bicycles (e-bikes) are established urban micromobility modes, and children and adolescents form a growing share of riders. This narrative review synthesises peer-reviewed evidence on e-scooter and e-bike injuries in patients aged 18 years or younger, covering epidemiology, mechanisms, injury patterns, clinical outcomes, comparisons with conventional devices, and prevention. Each source was classified as dedicated paediatric evidence, mixed-age evidence with extractable paediatric results, adult or mixed-age evidence used only for context, or an adult-dominated systematic review, so that the basis of every claim is explicit. The reported burden has risen across national surveillance data and single-centre series, and one population-adjusted analysis found injury rates increasing by 293% for e-bikes and by 88% for powered scooters between 2019 and 2022. Most studies lack exposure denominators, so exposure-adjusted paediatric risk remains poorly quantified and rising counts cannot be equated with rising risk. Injuries affect adolescent boys disproportionately and peak at ages 11 to 14 years. Extremity and soft-tissue injuries predominate, while a clinically important minority sustain traumatic brain injury, craniofacial and dental trauma, or severe multisystem injury. Low helmet use and higher device speeds are consistently associated with more severe outcomes, although observational designs cannot establish causal effects and device type is confounded with rider age and road exposure in every available paediatric comparison. Powered devices are associated with greater severity than non-powered counterparts. Speed limitation and helmet promotion appear promising, but paediatric-specific effectiveness evidence remains limited. Prospective paediatric research incorporating exposure denominators is the priority.

## 1. Introduction

Electric scooters (e-scooters) and electric bicycles (e-bikes) have rapidly become established features of urban micromobility, valued for their low cost, convenience, and perceived environmental benefits [1,2]. Following the large-scale expansion of shared, dockless e-scooter programmes into the ride-sharing market from 2017 onwards, ridership has grown steeply across North America, Europe, Asia, and Oceania [1,3]. Children and adolescents now constitute a substantial and increasing proportion of riders, frequently operating these devices for both recreation and short-distance commuting, often below the minimum ages recommended by manufacturers and regulators [3,4].

This rapid adoption has been paralleled by a sustained rise in device-related trauma presenting to emergency departments (EDs) worldwide, a trend so pronounced that some authors have described e-scooter injuries as a “new epidemic” in orthopaedic and trauma practice [2]. National surveillance data from the United States demonstrate a marked increase in e-scooter-related injuries and hospital admissions between 2014 and 2018 [1], and paediatric-specific analyses confirm that the burden among children and adolescents has continued to climb over the subsequent decade [4,5]. Comparable increases have been documented in dedicated paediatric and trauma-centre series across Europe and the Middle East, underscoring the global nature of the problem [6,7,8].

Paediatric micromobility injuries are shaped by developmental, behavioural, and environmental factors that distinguish them from adult injury patterns. Immature motor coordination, incomplete cognitive appraisal of risk, susceptibility to peer influence, limited road-safety experience, and infrequent use of protective equipment combine to place young riders at heightened risk of serious injury [3]. E-scooters and e-bikes reach substantially higher speeds than the manually propelled scooters and bicycles they resemble, and the additional kinetic energy this entails is a biomechanically plausible contributor to the more severe injury patterns reported, including traumatic brain injury, craniofacial and dental trauma, and displaced fractures requiring operative management [3,9]. Low helmet use among young riders is consistently associated with head and maxillofacial injury, although the observational designs on which this rests cannot quantify an independent causal effect [9,10,11].

The reported spectrum of paediatric e-scooter and e-bike trauma is broad, ranging from minor soft-tissue lacerations and contusions to life-threatening intracranial haemorrhage, solid-organ injury, and multisystem polytrauma [11,12]. A considerable proportion of affected children require hospital admission, surgical intervention, or intensive care, and a small but non-negligible number sustain potential long-term impairment or die [4,12]. Collectively, these injuries impose a significant and rising burden on paediatric emergency, surgical, and rehabilitation services [3,4].

Despite a rapidly expanding body of literature, the evidence base concerning paediatric e-scooter and e-bike injuries remains heterogeneous, comprising single-centre retrospective series, national database analyses, and injury-specific systematic reviews that vary in case definitions, age thresholds, and device classification [5,10,11]. A comprehensive synthesis focused specifically on children and adolescents is therefore warranted to inform clinicians, policymakers, and injury-prevention efforts. The aim of this narrative review is to consolidate the currently available peer-reviewed evidence on e-scooter and e-bike injuries in the paediatric population (≤18 years), addressing epidemiology and incidence trends, mechanisms and risk factors, anatomical injury patterns, clinical management and outcomes, comparisons with conventional devices, and prevention strategies, and to identify priorities for future research.

## 2. Materials and Methods

### 2.1. Review Design and Reporting Framework

This is a narrative review, written to provide an integrative account of a rapidly expanding and methodologically heterogeneous literature. Its conduct and reporting follow the Scale for the Assessment of Narrative Review Articles (SANRA), which addresses justification of the importance of the topic, statement of aims, description of the literature search, referencing, and the presentation of evidence and data [13].

The search and selection process is reported in structured detail, because transparency about how evidence was identified and weighted is what allows a narrative synthesis to be appraised. Section 2.2, Section 2.3, Section 2.4, Section 2.5 and Section 2.6 set out the search architecture, the date on which the databases were last searched, the eligibility criteria, the operational definitions of each device category, the two-stage screening process, the four-tier framework by which every source was classified according to how directly it bears on children, and the six credibility criteria used to weight evidence. The complete search strings are provided in Supplementary Table S1, the search and selection structure is shown schematically in Supplementary Figure S1, and the evidence-classification and weighting matrix for every included source is given in Supplementary Table S2. The scope, eligibility criteria, device definitions, and evidence-categorisation framework were specified in writing before screening commenced and were not altered thereafter, although no protocol was registered.

### 2.2. Search Strategy

Four electronic sources were searched, namely PubMed/MEDLINE, Scopus, the Web of Science Core Collection, and Google Scholar. The search was constructed from three concept blocks combined with the Boolean operator AND, with terms within each block combined using OR. Block 1 captured the device and included the terms “electric scooter”, “e-scooter”, “powered scooter”, “standing scooter”, “motorised scooter”, “electric bicycle”, “e-bike”, “pedelec”, and “micromobility”. Block 2 captured the outcome and included injury, trauma, fracture, concussion, accident, crash, and wound. Block 3 captured the population and included paediatric, pediatric, child, children, adolescent, teenager, and youth, supplemented by the controlled-vocabulary terms Child, Adolescent, and Infant in databases that support them. Truncation was applied to word stems and phrase searching to multi-word device terms. For Google Scholar, which does not support the same field syntax, a simplified version of the same three blocks was used and screening was restricted to the first 200 records ranked by relevance.

Searches were run from database inception to 30 June 2026, which was the date on which all four databases were last searched. The reference lists of all included articles and of relevant reviews were hand-searched, and forward citation tracking was performed on the most frequently cited articles. Because Block 3 would exclude mixed-age studies that carry no paediatric indexing terms, a parallel search omitting Block 3 was also run and screened both for extractable age-stratified data and for studies serving as indirect contextual evidence in the sense defined in Section 2.5.

### 2.3. Eligibility Criteria and Operational Definitions

Studies were eligible if they reported original clinical, epidemiological, or outcome data on e-scooter or e-bike injury in a population that included children or adolescents, or if they presented age-stratified data from which paediatric findings could be extracted. Case reports, case series, cross-sectional studies, retrospective and prospective cohorts, national injury-surveillance analyses, and systematic reviews were all eligible. Articles were restricted to those published in English. Excluded were studies reporting exclusively on adults with neither extractable paediatric data nor a contextual role as defined in Section 2.5, non-peer-reviewed sources, and editorials or commentaries without primary data.

The paediatric population was defined operationally as age of 18 years or younger, so that participants aged exactly 18 years were eligible. Where a source study applied a different upper bound, most commonly under 18 years or under 16 years, the study was retained and its own threshold was recorded during extraction and taken into account when interpreting its results, rather than harmonised to ours. No attempt was made to re-derive estimates to a common age band, because individual-level data were not available. Age thresholds are therefore not uniform across the studies summarised here, and this is a source of heterogeneity that should be borne in mind whenever proportions are compared between studies.

Device categories were defined as follows. A standing e-scooter is a two-wheeled powered device ridden from a standing platform with handlebars. A seated powered scooter carries a saddle and is otherwise comparable. A pedal-assist e-bike, or pedelec, provides motor assistance only while the rider pedals and, in most jurisdictions, withdraws assistance at 25 km/h. A throttle e-bike provides propulsion independent of pedalling. A speed pedelec provides pedal assistance to approximately 45 km/h and is legally classified as a moped in many jurisdictions. An e-moto is a heavier throttle-driven device resembling a motorcycle that is nevertheless often sold and ridden as though it were a bicycle. Many source studies, in particular analyses of national surveillance data, do not distinguish these categories and report a combined powered-micromobility or powered-scooter category. Where the device could not be resolved, the study is labelled as combined or unspecified in Table 2 and Supplementary Table S2, and its findings are not attributed to either device in the device-specific subsections.

### 2.4. Study Selection

Records retrieved from each database were exported and deduplicated by digital object identifier and, thereafter, by title, first author, and year for records lacking a digital object identifier. Titles and abstracts were screened independently by two authors. Full texts of records passing this stage were retrieved and assessed independently against the eligibility criteria by the same two authors. Disagreement at either stage was resolved by discussion, and where discussion did not produce agreement, a third senior author adjudicated. Reasons for exclusion at the full-text stage were recorded and applied consistently by both screening authors.

### 2.5. Evidence Categorisation and Appraisal

A recurring difficulty in this literature is that much of the granular evidence on mechanism, alcohol involvement, cost, and intervention effect derives from adult or mixed-age cohorts, whereas the question of interest is paediatric. Excluding such evidence would leave several clinically important domains unaddressed, whereas blending it silently with paediatric data would overstate the security of paediatric inference. Each included source was therefore assigned to one of four evidence categories.

Category A comprises dedicated paediatric studies, in which the entire study population is aged 18 years or younger. Category B comprises mixed-age studies from which paediatric results can be extracted directly, either as a reported subgroup or from age-stratified tables. Category C comprises mixed-age or adult studies used only as indirect contextual evidence, and for two purposes only, namely the predefined questions where paediatric data are absent, which are device biomechanics, alcohol involvement, health-economic burden, and the effect of population-level interventions, and the description of the wider all-age literature within which the paediatric evidence sits. Category D comprises systematic reviews and meta-analyses whose included populations are predominantly adult. Every study in Table 2 and Supplementary Table S2 carries its category, and every substantive claim in the sections that follow is attributable to a category through its citations. Statements resting on Category C or Category D evidence are identified as such in the text, and the Abstract and Conclusions reserve definitive paediatric wording for findings supported by Category A or Category B evidence.

Formal risk-of-bias instruments designed for randomised trials or for aetiological cohort studies map poorly onto descriptive injury-surveillance analyses and single-centre trauma series, which constitute most of this literature. Rather than apply an ill-fitting scored instrument, each study was appraised against six credibility criteria, namely whether the population was assembled prospectively or retrospectively, whether the case definition for device type was explicit, whether an exposure denominator was available, whether injury severity was defined using a validated scale, whether helmet status was ascertained systematically or from chart review, and whether reported comparisons were adjusted for confounding. These criteria were used to weight evidence in the narrative synthesis, and the resulting interpretative weight is recorded for every source in Supplementary Table S2 together with its reason. Greater interpretative weight was given to studies with an explicit device case definition, a defined denominator, and adjusted comparisons, and correspondingly less to small selected referral series and to studies in which device type was inferred from free-text triage narratives.

### 2.6. Approach to Synthesis

Given the substantial heterogeneity in design, case definition, age threshold, device classification, and severity metric across the available evidence, a formal quantitative meta-analysis was not undertaken. Findings are synthesised narratively and tabulated to permit comparison of study populations, device types, injury patterns, and outcomes. Where a claim is supported by studies that disagree, the disagreement is reported rather than reconciled. Where systematic reviews and their component primary studies both appear in this review, the primary studies are cited for study-level detail and the review is cited for its pooled estimate alone, and the possibility that the same patients contribute to both is noted at the point of use. Pooled proportions drawn from predominantly adult syntheses are presented as indirect evidence and are not offered as paediatric frequency estimates.

## 3. Epidemiology and Incidence Trends

### 3.1. International Trends in Reported Injuries and Emergency Presentations

The number of reported e-scooter and e-bike injuries has risen sharply since the commercial introduction of shared micromobility services. In the United States, the National Electronic Injury Surveillance System (NEISS), a probability sample of approximately 100 emergency departments that yields weighted national estimates of emergency-department-treated product-related injury, recorded an increase in e-scooter-related injuries from 4582 in 2014 to 14,651 in 2018, accompanied by a parallel rise in hospital admissions [1]. These are national estimates of emergency-department-treated injury rather than counts of all injuries, and they exclude children who did not seek emergency care or who were managed outside hospital. Paediatric-specific analyses of the same national dataset confirm that this upward trajectory has continued, with e-scooter injuries in patients aged 18 years or younger increasing over the decade from 2014 to 2023, particularly following the onset of the COVID-19 pandemic [5].

More recent national analyses extend this picture and, importantly, supply the first population-adjusted estimates. Between 2017 and 2022, NEISS-derived national estimates rose from 8566 to 56,847 annually for e-scooter injuries and from 751 to 23,493 annually for e-bicycle injuries, giving totals of 189,517 e-scooter and 45,586 e-bicycle injuries across the six-year period, set against approximately 2.5 million conventional bicycle and 304,000 conventional scooter injuries [9,14]. A published correction subsequently revised two values in the first table of the scooter and bicycle comparison, and the corrected figures are the ones quoted here [15]. E-bicycle injuries alone increased approximately thirty-fold over the period, with an estimated 5462 associated hospitalisations [14]. The United States Consumer Product Safety Commission, in surveillance covering 2017 to 2024, recorded 533 micromobility-related deaths, rising from 5 in 2017 to 135 in 2024, of which 206 involved e-scooters, 310 involved e-bikes, and 17 involved self-balancing scooters, and independently estimated 155,200 emergency-department-treated e-bicycle injuries across the same eight-year period, of which 59,200 occurred in 2024 alone [16]. Most importantly for interpretation, an analysis of 1,933,296 estimated micromobility injuries between 2019 and 2022 reported population-based rates rather than counts alone and found that these rates increased by 293.0% for e-bikes and by 88.0% for powered scooters [17]. Because that estimate carries a general-population denominator, it indicates that the rise in reported injury is not merely an artefact of population growth, although it still does not adjust for ridership. One caution applies to all of the United States estimates quoted above. Several of them are derived from the same national surveillance system over overlapping periods, and two of the paediatric analyses cited here draw on the same decade of records and were reported by the same research group, so these figures are not independent of one another. They should not be summed, and their agreement should not be read as independent corroboration of the same trend.

**Figure 1:**
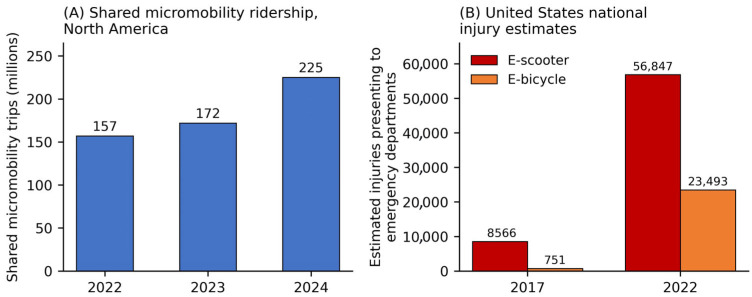
Growth in electric micromobility use and in reported injury. (**A**) Shared micromobility trips across North America, 2022 to 2024 [18]. (**B**) Weighted national estimates of e-scooter and e-bicycle injuries presenting to United States emergency departments in 2017 and in 2022 [9,15]. Intermediate years are not plotted because the cited sources do not report comparable annual values for every year, and the two panels therefore illustrate direction and magnitude rather than a fitted trend. Panel B values are estimates of emergency-department-treated injury and are not counts of all injuries, and they carry no exposure denominator, so they should not be read as injury rates per rider or per trip.

Exposure data are rarely linked to injury data within the same analysis, but they are now available for shared fleets in North America and provide essential context for the counts above. Shared micromobility trips rose from approximately 157 million in 2022 to 172 million in 2023 and to at least 225 million in 2024, an increase of 31% in a single year, across 415 cities and a deployed fleet of 333,000 vehicles, with 66% of trips taken on electric devices and e-scooters accounting for 48% of the fleet. Shared e-bike trips alone rose from 18.8 million in 2021 to 30.9 million in 2022 [18]. Private ownership has grown in parallel, with United States e-bicycle imports exceeding 1.1 million units in 2022 compared with 437,000 in 2020 [14]. Placing these figures beside the injury estimates above shows that ridership and reported injury have grown together, and that available data do not allow the two trends to be separated with confidence in most settings. Reported ridership and injury trends are summarised in Table 1 and illustrated in Figure 1.

This trend is not confined to North America. Following the introduction of shared e-scooter schemes, emergency departments in Europe, the Middle East, and Oceania have reported steep increases in device-related presentations [7,8]. A large single-centre Israeli series documented more than 3300 e-scooter injuries after shared services were introduced [8], while nationwide data from Finland demonstrated a rising incidence of e-scooter-associated injuries between 2019 and 2021 [7]. Dedicated paediatric series have mirrored these findings. An urban tertiary paediatric emergency department reported a 3.5-fold increase in powered-scooter and e-bike presentations over the study period, with a parallel rise in hospitalisations, surgical interventions, and severe trauma [4]. These reports are concentrated in high-income countries with established shared-fleet markets and well-developed emergency-care surveillance, and data from low- and middle-income settings are almost entirely absent, so the international picture assembled here should not be read as a global one.

Injuries from manually propelled scooters in children were described well before the advent of powered micromobility [20,21,22,23,24,25], but the electrification of these devices has been associated with a distinct escalation in both the frequency and severity of paediatric trauma [26]. Several authors have specifically implicated the COVID-19 pandemic period, during which recreational micromobility use among children increased, as a point of inflexion in paediatric injury trends [5,27].

### 3.2. Age and Sex Distribution

Across the paediatric literature, e-scooter and e-bike injuries display a consistent demographic signature, a pronounced male predominance and a peak in early-to-mid adolescence. In a national analysis derived from a probability sample of emergency departments, covering 2117 paediatric e-scooter injuries recorded in the United States between 2020 and 2024, boys accounted for approximately 70% of cases, and children aged 11–14 years represented the single largest age group at 38.3%, compared with 27.7% among those aged 6–10 years, 27.2% among those aged 15–17 years, and 6.7% among those aged 0–5 years [19].

This pattern is reproduced across single-centre and multicentre series, in which male proportions of 63–82% have been reported and mean ages typically cluster between 11 and 14 years [4,6,26,28]. A retrospective series of children hospitalised with e-scooter-related trauma reported a mean age of 11.3 ± 3.7 years and a male proportion of 81.5% [28], whereas a comparative Austrian study found that e-scooter riders were substantially older than users of non-electric scooters (mean 14.2 vs. 8.0 years), consistent with the greater speed and road exposure of powered devices [26]. Children and adolescents thus bear a substantial and rising share of the overall injury burden, with those aged 11–17 years alone accounting for roughly two-thirds of paediatric e-scooter injuries in national data [19]. Representative studies across settings, geographies, and study designs are summarised in Table 2.

Studies were selected for Table 2 according to four explicit rules, namely that the study contributed dedicated paediatric or extractable age-stratified data wherever such data existed, that it was among the larger studies available for its design type, that it added geographic diversity, and that it represented a distinct study design or a distinct anatomical or outcome domain. The table is therefore illustrative rather than exhaustive. Evidence category refers to the four-tier framework defined in Section 2.5, where A denotes a dedicated paediatric study, B a mixed-age study with extractable paediatric results, C a mixed-age or adult study used only as indirect contextual evidence, and D an adult-dominated systematic review. Helmet use is reported as stated in the source study, and NR denotes not reported. Where a percentage is given, it refers to patients in whom helmet status was documented rather than to all patients, unless the source stated otherwise. Age definitions differ between studies and are not harmonised here. The device classification, the evidence category, the manuscript sections in which each source is principally used, and the interpretative weight assigned to it are recorded for every included source in Supplementary Table S2.

**Table 2:** Summary of representative studies of electric scooter and electric bicycle injury in children, adolescents, and mixed-age populations.

Study (Year)	Country	Design	Population (*n*)	Helmet Use	Key Findings	Device, Category
Namiri et al. (2020) [1]	USA	National database (NEISS)	National, 2014–2018	NR	Injuries rose 4582 to 14,651; rising hospital admissions	E-scooter, C
Moati et al. (2025) [4]	Israel	Retrospective single-centre ED	1466 children	NR	3.5-fold rise in presentations, 14.7% admitted, 3 deaths (device attribution not reported in source)	E-scooter and e-bike, A
Schuller et al. (2025) [26]	Austria	Retrospective single-centre	633 children	NR	e-scooter riders older; more traffic accidents and severe head injury than non-electric	E-scooter vs. non-powered, A
Howard et al. (2026) [19]	USA	National database (NEISS)	2117 children, 2020–2024	NR	Male 70%; ages 11–14 largest group; racial/ethnic disparities	E-scooter, A
Abu-Kishk et al. (2025) [28]	Israel	Retrospective single-centre	103 hospitalised children	62.5%	Orthopaedic 70.9%; PICU 11.6%; helmet protective against head injury	E-scooter, A
Trivedi et al. (2019) [29]	USA	Prospective cohort (2 EDs)	249 injured riders	<5%	Early prospective series, head injury common, helmet use below 5%	Standing e-scooter, C
Weiss et al. (2025) [30]	USA	National database (NEISS)	National paediatric	NR	Lower arm and wrist most fractured; radius commonest bone	E-scooter, A
Buyukceran et al. (2023) [31]	Turkiye	Retrospective single-centre	99 (49 paediatric)	NR	Children upper-extremity; adults lower-extremity fractures	E-scooter, B
Kleinertz et al. (2023) [32]	Germany	Retrospective single-centre	268	<1%	Head/face impact 58% (84% if intoxicated); non-rider injuries described	E-scooter, C
Cirillo et al. (2025) [33]	Italy	Retrospective tertiary ED	411	NR	Injury patterns and modifiable risk factors (alcohol, helmet)	E-scooter, B
Foglar et al. (2025) [34]	Hungary	Retrospective single-centre	1938 (mixed micromobility)	NR	Comparative injury patterns and severity across device types	Combined micromobility, C
Suominen et al. (2022) [35]	Finland	Retrospective single-centre	104 e-scooter TBI	3.8%	71% intoxicated; helmet documented in 3.8%	E-scooter, C
Ruan et al. (2024) [36]	USA	National database (NEISS)	National ocular	NR	Orbital fractures ~5-fold higher than non-powered scooters	E-scooter vs. non-powered, C
Pakarinen et al. (2023) [37]	Finland	Population-based natural experiment	Population-based	NR	Speed and night-time limits approximately halved injury incidence	E-scooter, C

ED, emergency department; NEISS, National Electronic Injury Surveillance System; NR, not reported; PICU, paediatric intensive-care unit; TBI, traumatic brain injury. Studies were selected to illustrate the range of settings, geographies, and study designs and are not exhaustive.

### 3.3. Sociodemographic Disparities

Emerging evidence indicates that the burden of paediatric e-scooter injury is not distributed evenly across sociodemographic groups. In the United States national analysis, Black (16.0%) and Hispanic (15.7%) children accounted for a disproportionately greater share of e-scooter injuries relative to their representation among all paediatric injuries. The comparison denominator here is all paediatric injuries rather than paediatric micromobility exposure, and that distinction matters. A greater share among injured children does not establish a higher injury rate per rider, per trip, or per kilometre travelled, because the observed difference could equally reflect the geographical distribution of shared fleets, urbanicity, the age structure of the populations concerned, patterns of transport use, or differential access to emergency care and differences in coding. In adjusted analyses, however, these racial and ethnic differences were not associated with a significantly greater likelihood of hospitalisation, which is consistent with, although it does not demonstrate, the observed disparities reflecting differences in exposure rather than in injury severity [19].

The mechanisms set out in the following sentence are advanced as hypotheses rather than as findings because none of them was examined directly in the cited analysis. They are likely to be multifactorial and to encompass differential access to safe, dedicated riding infrastructure, variation in the enforcement of age and helmet regulations, and socioeconomic factors influencing supervision and protective-equipment use [19]. These observations support the design of injury-prevention strategies that are equitable and attentive to environmental and structural determinants of risk. We note, however, that the cited analysis did not test a socioeconomic comparison directly, so we do not assert that lower-income communities bear a disproportionate injury burden. These findings also derive almost entirely from data collected in the United States and warrant validation in other healthcare settings before they are generalised.

### 3.4. Early and Frequently Cited Studies and International Cohorts

The paediatric evidence base sits within a broader, rapidly expanding literature on e-scooter trauma across all ages, the trajectory of which informs paediatric interpretation. An early prospective characterisation of standing e-scooter injury by Trivedi and colleagues first drew clinical attention to the scale of the problem and to the low prevalence of helmet use among injured riders [29]. Early single-centre series from the United States, New Zealand, and Australia documented the abrupt appearance of e-scooter trauma following the roll-out of shared services, with one trauma service describing e-scooter injury as an emerging epidemic [38,39].

International cohorts have since confirmed the global and escalating nature of the phenomenon. Large single-centre and multicentre series from Germany [32], Italy [33], Hungary [34], Lithuania [40], the United Kingdom [41,42], and Finland [43] consistently report rising presentations, a young and male-predominant case mix, low helmet use, and a substantial burden of extremity and craniofacial injury. A Hungarian level-1 trauma-centre study alone identified 1938 micromobility-injured patients over a single year [34], whereas national and registry analyses from the United States describe parallel increases in hospital admission and resource utilisation [44,45]. Although these cohorts are dominated by adults, children and adolescents form a consistent and growing subgroup within them [4,33].

### 3.5. Non-Rider and Bystander Injuries

The public-health footprint of e-scooters extends beyond riders to non-riders and bystanders. Pedestrians, particularly older adults, are injured by tripping over improperly parked devices or in collisions with riders on shared footpaths [32,46]. In one of the largest European cohorts, non-rider casualties included cyclists who collided with e-scooter riders and pedestrians with a median age of 61 years who had stumbled over parked scooters [32]. Although children are more often injured as riders than as bystanders, the shared use of pavements and cycle lanes means that paediatric pedestrians are also exposed to collision risk [47,48].

National trauma-registry data that combine electric bikes and motorised scooters have documented a rising toll of road-traffic casualties, including vulnerable non-motorised road users [47]. These observations reinforce that prevention strategies, particularly parking regulation, speed limitation, and separation of micromobility from pedestrian space, protect not only young riders but the wider community [32,46].

### 3.6. The COVID-19 Pandemic and Micromobility Injury

The COVID-19 pandemic exerted a complex influence on micromobility injury. Early rental-programme studies had already established a steep rise in presentations after shared-service launches, including a six-fold monthly increase in one series and comparable cohorts from North America and Scandinavia [8,49,50,51]. Public-health lockdowns then temporarily reduced ridership and injury presentations, with several centres documenting a marked fall in accidents during the strictest restriction periods [2,52]. Paediatric analyses specifically identify the pandemic period as an inflexion point after which e-scooter and e-bike injuries in children rose steeply [5,27].

As restrictions eased, micromobility use rebounded sharply, and follow-up studies reported injury numbers exceeding pre-pandemic levels [53]. Contributing factors included a shift away from public transport towards individual micromobility and increased recreational riding by children confined to their neighbourhoods [54]. Analyses of other active-transport modes during the pandemic, including a national rise in cyclist injuries despite reduced vehicle travel, corroborate a broad pandemic-era increase in vulnerable-road-user trauma [55].

## 4. Mechanisms and Risk Factors

The mechanisms and risk factors underlying paediatric e-scooter and e-bike trauma reflect a combination of device characteristics, rider behaviour, and environmental exposures. Although much of the granular mechanistic data derives from mixed-age and adult cohorts, the principal contributory factors, including falls, collisions, low rates of helmet use, and higher travelling speeds, are directly relevant to children and adolescents, in whom immature motor control and risk appraisal further amplify their effect [3,19,56].

### 4.1. Mechanisms of Injury

The predominant mechanism of injury across the e-scooter literature is a simple fall from the device, followed by collisions with fixed objects, kerbs, or motor vehicles [56,57]. Falls frequently pitch the rider forwards over the handlebars, producing a characteristic pattern of head, facial, and upper-extremity injuries as the outstretched arms absorb the impact [32]. In one of the largest European cohorts, riders sustained an impact to the head or face in 58% of crashes [32].

Small wheel diameter, a high centre of gravity, and rapid acceleration are widely held to render e-scooters susceptible to instability when encountering uneven road surfaces, potholes, or tram tracks [57,58]. It should be stated plainly that this account is a mechanistic interpretation rather than an experimentally demonstrated one. It rests on the consistency of reported crash circumstances in observational series and on general principles of vehicle dynamics, and we are not aware of controlled biomechanical or track-based studies in child riders that have measured these parameters directly. The explanation is therefore offered as plausible inference and not as established fact. Collisions with motor vehicles, although less frequent than isolated falls, are associated with disproportionately severe injuries and have been specifically characterised in national surveillance data [59]. Riding two abreast or carrying a passenger, common among adolescents, further destabilises the device and is recognised as an avoidable contributory factor [56].

### 4.2. Helmet Use and Protective Equipment

Consistently low rates of helmet use are among the most frequently cited and most modifiable risk factors for serious e-scooter injury [57]. Helmet use among e-scooter riders is reported to be substantially lower than among cyclists, with some series documenting helmets in fewer than 5% of injured riders [32]. In dedicated paediatric cohorts, helmet use is variable but often incomplete. One hospitalised paediatric series documented helmet use in 62.5% of cases [28], a figure markedly higher than that reported in most other series and one that should be read in the light of its denominator, since it refers to children in whom helmet status was recorded rather than to all children admitted. Reporting of helmet status is incomplete in most series, and percentages are therefore not comparable unless the denominator is stated. National data report the head as the most frequently injured single coded body region in young riders [19]. This is compatible with the predominance of musculoskeletal injury described in Section 5.1 because the musculoskeletal category aggregates fractures, dislocations, strains, and sprains across several body regions, whereas the head is a single region.

The protective effect of helmets against head and maxillofacial injury is well established for cycling and is increasingly supported for powered micromobility. A systematic review and meta-analysis of helmet use in bicycle and scooter accidents found that helmet use was associated with a lower risk of maxillofacial injuries [10]. The low uptake of helmets among children and adolescents, attributed to inconvenience, unavailability of rental devices, and perceived low risk, therefore represents a key target for prevention [56].

### 4.3. Speed, Riding Behaviour, and Environmental Factors

Achievable speed is one of the clearest measurable differences between powered and conventional micromobility. E-scooters and e-bikes readily attain travelling speeds of 25–30 km/h, and the greater kinetic energy involved is a plausible contributor to the more severe injuries reported in riders of powered devices. The comparative studies available are, however, observational, and their powered-device groups also differ in rider age, road exposure, and riding context, so the contribution of speed itself cannot be isolated [26]. Evidence on speed restriction comes from natural experiments rather than from randomised designs. A natural-experiment study in Helsinki reported that injury rates fell after the introduction of speed and night-time usage restrictions, from 19 to 9 per 100,000 rides, which is one of very few estimates in this literature to carry a ride-based denominator [37]. Attribution of that decline specifically to the restrictions remains provisional, because fleet size, seasonality, enforcement intensity, and concurrent measures were not controlled.

Temporal and environmental factors also modulate risk. E-scooter injuries cluster at night and at weekends, periods associated with recreational riding, impaired visibility, and alcohol consumption [35,60]. Inexperience is a further important determinant, with injuries frequently occurring during a rider’s first few uses of a device [57]. For children and adolescents, riding on roads shared with motor traffic rather than on dedicated lanes increases exposure to high-energy collisions [19].

### 4.4. Alcohol and Substance Use

Alcohol and, to a lesser extent, recreational drug use are strongly associated with e-scooter trauma in adult and older-adolescent populations [32,57]. In adult tertiary-hospital series of e-scooter-related traumatic brain injury, the majority of patients are intoxicated at the time of injury and helmet use is almost universally absent [35]. Riders under the influence of alcohol are considerably more likely to fall on the head or face. In one European cohort, 84% of intoxicated riders sustained a head or facial impact, compared with 58% of riders overall [32].

Although acute alcohol intoxication is of limited relevance to younger children, it is an important and growing concern among older adolescents, in whom it compounds inexperience and low helmet use [56,57]. Systematic reviews consistently identify substance use, helmet avoidance, and rider inexperience as the dominant modifiable contributors to severe e-scooter injury [57].

### 4.5. Behavioural and Human Factors

Behavioural and developmental factors are central to paediatric micromobility risk. Early adolescence is characterised by heightened risk-taking, susceptibility to peer influence, less-developed motor skills, poorer hazard judgement, and limited road-safety experience, a combination that places children aged 11 to 14 years at particular risk [3,19]. Attitudinal studies of e-scooter users confirm widespread risk-taking behaviours, including riding without helmets, tandem riding, and the use of privately owned devices modified for higher speeds [61].

These behavioural determinants have direct implications for prevention, since attitudes and habits formed in adolescence shape long-term road-user behaviour. Structural and legislative measures have been followed by measurable reductions in injury, whereas educational interventions in this field have not been evaluated against injury outcomes, so combined approaches are advocated on that basis rather than on a head-to-head comparison [62,63]. Evidence on the determinants of protective-equipment use indicates that convenience, social norms, and enforcement all influence helmet uptake [61,64]. Tailoring safety messaging to the developmental characteristics of young riders is therefore a priority [3].

## 5. Injury Patterns and Anatomical Distribution

Paediatric e-scooter and e-bike trauma affects virtually every body region, but the distribution is dominated by extremity and soft-tissue injuries, with head and facial trauma representing the most clinically significant subset [19,28]. The relative frequency of each pattern varies with case mix, mechanism, and whether a study captures emergency-department attendances or hospital admissions, but several consistent themes emerge across the literature [4,6,28].

### 5.1. Overall Anatomical Distribution

In the largest national paediatric dataset, which is derived from sampled emergency departments and therefore describes emergency-department-treated injury, musculoskeletal injuries, fractures, dislocations, strains, and sprains, accounted for 40.4% of e-scooter injuries and soft-tissue injuries for a further 36.7% [19]. Hospital-based paediatric series, which are enriched for more severe presentations, report an even greater preponderance of orthopaedic injury. In one cohort of 103 hospitalised children, orthopaedic injuries constituted 70.9% of cases, followed by general paediatric surgical injuries (10.7%) [28]. The most commonly injured regions are the limbs, head and neck, thorax, and abdomen, with limb fractures most frequently involving the radius, ulna, tibia, femur, and fibula [28,65,66].

Injury severity spans the full spectrum. Although the majority of children sustain minor injuries amenable to outpatient management, a substantial minority present with polytrauma, and older children are more likely than younger children to sustain multiple injuries [6]. The following subsections consider the principal anatomical patterns in turn. One caveat applies throughout. The categories used in the source studies, namely head, facial, ocular, dental, fracture, and soft-tissue injury, are not mutually exclusive, and a single child may contribute to several of them. Proportions reported by anatomical region, therefore, do not sum to 100% and should not be treated as a partition of the injured population. Where a source study stated whether its categories were exclusive, this is noted at the point of use.

### 5.2. Traumatic Brain Injury and Head Trauma

Traumatic brain injury (TBI) is the most serious commonly encountered consequence of paediatric micromobility trauma and a leading contributor to morbidity and mortality [28]. Reported rates of head injury among e-scooter riders vary widely, between approximately 15% and 40% of all injuries [35]. This nearly threefold spread is itself informative and deserves comment rather than simple restatement. It arises from at least five sources. Case definitions differ, since some series count any injury above the neck while others count only intracranial injury or only injuries meeting a severity threshold. Study settings differ, and emergency-department cohorts, trauma-registry cohorts, and neurosurgical referral cohorts sample progressively more severely injured patients. Imaging practice differs between centres and over time, so the ascertainment of intracranial injury is not uniform. Age composition differs, and the anatomical distribution of injury changes with rider age. Finally, helmet prevalence differs markedly between settings and between shared and privately owned devices. Reported proportions should therefore be compared only where case definition, setting, and imaging practice are broadly comparable, and the range should not be reduced to a single pooled figure. In a hospitalised paediatric cohort, head trauma was diagnosed in 14.6% of children, of whom 46% had intracranial haemorrhage and 26% sustained a skull fracture [28]. Not wearing a helmet was significantly associated with head trauma, and nearly half of the unhelmeted head-injury cases involved intracranial haemorrhage [28].

Greater head-injury severity has been reported in association with the higher speeds attainable on powered devices. A comparative study demonstrated that adolescent e-scooter riders sustained severe head injuries more often than riders of non-electric scooters [26]. The term severe head injury is not used consistently across the studies cited in this review. Depending on the source, it denotes a Glasgow Coma Scale score below 9, an Abbreviated Injury Scale score for the head of 3 or above, any radiologically confirmed intracranial injury, or an injury requiring neurosurgical intervention. We report each study’s own definition where it was stated, and readers should not assume that severe head injury denotes the same entity across studies. The clinical spectrum ranges from mild concussion to skull fracture and intracranial haemorrhage, and TBI in childhood carries the potential for long-term neurological, cognitive, and psychiatric sequelae [28].

### 5.3. Craniofacial and Dental Injuries

The face is exposed during the characteristic forward fall over the handlebars, and craniofacial injuries are correspondingly frequent [11,67]. A systematic review of e-scooter craniofacial trauma encompassing 539 patients found that mechanical falls accounted for 72.4% of injuries, that middle-third fractures were the most common pattern among the 398 fractures recorded in those patients (58.3% of fractures), and that soft-tissue injuries (58.3% of patients), dental injuries (32.9%), traumatic brain injuries (17.6%), and ophthalmological injuries (20.6%) were all well represented. Surgical management was required for 43.3% of fractures [11]. Nasal fractures are the most frequently reported facial fracture, followed by orbital-wall and zygomaticomaxillary-complex fractures [67]. Comparative studies consistently report a distinct fracture distribution in e-scooter riders relative to cyclists, with mandibular and midface fractures predominating, and describe a COVID-era surge in such injuries [68,69,70]. Retrospective series from multiple European centres confirm that e-scooter maxillofacial fractures frequently require operative fixation [71,72,73,74].

Dental and dento-alveolar trauma is a distinctive feature of e-scooter injury. Crown-root fractures and tooth avulsions occur at higher reported rates in e-scooter accidents than in other trauma mechanisms, and such injuries are associated with challenging treatment protocols and a poorer prognosis [75]. Because craniofacial and dental injuries frequently coexist with occult brain trauma, a complete craniofacial and oral examination is recommended when assessing children after e-scooter accidents [76]. The demonstrated protective effect of helmets against maxillofacial injury reinforces the case for their use in this age group [10].

### 5.4. Extremity and Orthopaedic Injuries

Extremity fractures are the single most common reason for hospital admission and surgery following paediatric e-scooter trauma [28,31]. The mechanism, falling onto an outstretched hand from riding height, produces a marked predominance of upper-extremity injury in children [31,77]. In a comparative study of e-scooter fractures, 59.5% involved the upper extremity and 27.2% the lower extremity, and the paediatric group sustained predominantly upper-extremity injuries, whereas adults more often sustained lower-extremity injuries [31].

National paediatric data corroborate this distribution. Among e-scooter fractures in children, the lower arm (27.9%) and wrist (26.3%) were the most commonly fractured body parts, and the radius was the most commonly fractured bone (33.8%), followed by the clavicle (8.7%) [30]. In a hospitalised paediatric series, limb fractures were identified in 67% of patients, of which upper-limb fractures accounted for approximately half and lower-limb fractures for about one-third [28]. A considerable proportion of these fractures require operative fixation, and orthopaedic procedures constitute the majority of surgical interventions in children admitted with e-scooter trauma [28,78].

### 5.5. Truncal, Abdominal, and Spinal Injuries

Truncal, abdominal, and spinal injuries are less frequent than extremity and head trauma but are clinically important because of their potential severity [6,28]. Chest and abdominal trauma range from osseous and soft-tissue contusions to fractures of the thorax or vertebrae and lacerations of solid abdominal or thoracic organs [6]. In a large paediatric scooter series, trunk injuries accounted for approximately 5% of cases, and a small proportion of these involved lacerations of abdominal organs [6].

Abdominal and thoracic solid-organ injury, although uncommon, can be life-threatening and may necessitate operative intervention or intensive-care admission [28]. Spinal injuries are infrequently reported in the paediatric e-scooter literature but remain a recognised consequence of high-energy collisions, particularly those involving motor vehicles [59]. Neurosurgical case series describe cervical-spine fractures, vertebral-body compression fractures, and central cord syndrome in association with e-scooter and e-bike trauma, and severe-traumatic-brain-injury cohorts include concomitant spinal injury, including in pedestrians struck by e-scooters [79,80,81,82]. Spinal trauma has been reported in as many as one in six patients in a dedicated neurosurgical series. That proportion should not be read as the frequency of spinal injury among injured child riders generally. Neurosurgical referral cohorts are assembled from patients already selected for suspected central nervous system injury, so the denominator is enriched for severe cases and the figure will overestimate the true frequency in an unselected paediatric population [83]. The appropriate inference is awareness of the possibility of spinal injury after high-energy mechanisms, assessed according to established paediatric trauma criteria, rather than a lowered threshold for spinal immobilisation based on device type alone. The relative rarity of these injuries, combined with the predominance of single-centre data, means that their true incidence in children is likely under-characterised [28].

### 5.6. Ocular and Periocular Injuries

Ocular and periocular trauma is an increasingly recognised component of e-scooter injury that is frequently underestimated when injuries are classified by broad body region [36,84,85]. The forward trajectory of a fall over the handlebars exposes the orbit and periorbital soft tissues to direct impact, producing eyebrow and eyelid lacerations, periorbital contusions, orbital fractures, and corneal abrasions [36,84]. In a national analysis of eye and orbit injuries, e-scooter-related ocular injuries increased markedly over the study period, and orbital fractures were reported more frequently among e-scooter riders than among non-powered-scooter riders [36].

The magnitude of this excess risk is striking. E-scooter riders have been reported to be several-fold more likely to sustain orbital fractures than riders of non-motorised scooters [86]. Vision-threatening complications, including retrobulbar haemorrhage requiring emergency lateral canthotomy and cantholysis, are described, although globe rupture is rare [84]. Because ocular injuries frequently coexist with maxillofacial fractures and intracranial trauma, formal ophthalmological assessment should be considered in children presenting with periorbital injury where examination findings or visual symptoms indicate it, although the co-occurrence data reviewed here have not been evaluated as a referral criterion [84,87].

### 5.7. Evidence from Systematic Reviews and Meta-Analyses

The consistency of these anatomical patterns across heterogeneous studies is confirmed by several systematic reviews and meta-analyses. A proportional meta-analysis of emergency-department presentations, drawing on 149 studies that met its inclusion criteria, of which 68 were eligible for quantitative synthesis, estimated pooled frequencies of head and facial injury of 42.1%, upper-extremity injury of 40.1%, and extremity fracture of 25.7%, with severe traumatic brain injury in 2.3% and severe overall injury (Injury Severity Score ≥ 16) in 2.8% of patients (Table 3) [57]. That synthesis reported the number of contributing studies for each outcome rather than an outcome-specific participant count, and those numbers ranged from 16 studies for extremity fracture to 24 for head and facial injury. A separate systematic review of 34 studies encompassing 5702 injuries similarly identified falls as the dominant mechanism (74.4%), followed by collision with a vehicle (10.2%) [88]. The pooled values in Table 3 are indirect evidence from predominantly adult or mixed-age populations and are not paediatric frequency estimates. The number of contributing studies and participants differs between outcomes, and the source syntheses did not report an outcome-specific denominator for every parameter, so no pooled value should be read as though it rested on the full sample. Categories are not necessarily mutually exclusive, since a single patient may contribute to more than one anatomical category.

A proportion meta-analysis of 25 studies comprising 5387 patients described two dominant phenotypes, a mildly injured group with predominantly upper-limb trauma and a severely injured group with predominant head trauma [89]. Comparative meta-analysis of powered vs. non-powered devices supports the conclusion that electrification is associated with a shift towards more severe injury patterns [90,91]. These syntheses are dominated by adult data. They therefore constitute indirect evidence in the sense of Category D defined in Section 2.5, and their pooled proportions are not paediatric frequency estimates and should not be quoted as such. They remain useful because their convergent findings indicate which anatomical patterns are robust to differences in setting and case mix, but the magnitude of each proportion in children has still to be established by dedicated paediatric studies [57,89]. A further caution concerns overlap. Several of the primary studies cited elsewhere in this review also contribute patients to these syntheses, so the same patients may be represented more than once in the evidence summarised here. We have accordingly cited the primary studies for study-level detail and the syntheses for their pooled estimates alone, and we have not summed proportions across the two.

## 6. Clinical Management and Outcomes

The clinical trajectory of paediatric e-scooter and e-bike trauma ranges from brief emergency-department attendance for minor injuries to prolonged multidisciplinary inpatient care for polytrauma and severe traumatic brain injury [4,19,28]. Outcomes are generally favourable in aggregate but are punctuated by a minority of children who sustain life-changing or fatal injuries [19,28].

### 6.1. Emergency Presentation and Acute Management

Most children injured on e-scooters and e-bikes present to the emergency department with isolated extremity or soft-tissue injuries that can be managed on an outpatient basis [19]. Initial assessment follows standard paediatric trauma principles, with particular vigilance for occult head, cervical-spine, and intra-abdominal injury, given the mechanism and the frequent coexistence of craniofacial trauma with brain injury [28,76]. Intracranial haemorrhage has been reported disproportionately often in unhelmeted children who sustain head impact [28]. That observation describes a reported injury pattern and is not a diagnostic recommendation. Decisions about cranial imaging in children should follow validated paediatric head-injury decision rules, which weigh mechanism, symptoms, examination findings, age, and the established predictors of clinically important traumatic brain injury [92,93]. Helmet status is useful contextual information about the mechanism, but the evidence assembled here does not establish that it independently mandates imaging, and injury-frequency studies of this design do not validate diagnostic pathways.

Because dental and craniofacial injuries may signal concurrent intracranial trauma, a complete craniofacial and oral examination is recommended, and early involvement of maxillofacial, neurosurgical, and orthopaedic services is often required [11,76].

### 6.2. Hospitalisation, Surgery, and Intensive Care

Rates of hospital admission vary with case mix and health-system thresholds. In a nationally representative paediatric analysis, the weighted hospitalisation rate was 7.7% [19], whereas dedicated paediatric emergency-department series report higher admission proportions, for example, 14.7% in one urban tertiary cohort [4]. Among admitted children, the intensity of care can be considerable. Approximately half of hospitalised children in one series underwent a surgical procedure, predominantly orthopaedic fixation, and 11.6% required paediatric intensive-care admission [28].

Surgical intervention is common at the severe end of the injury spectrum. In a systematic review of craniofacial trauma, 43.3% of facial fractures required operative management [11]. Children requiring intensive care have longer hospital stays, and traumatic brain injury in particular may necessitate neurosurgical intervention, intracranial-pressure monitoring, and prolonged rehabilitation [94]. Reported hospital length of stay ranges from a single day to several weeks depending on injury severity and intensive-care requirement [94].

### 6.3. Mortality, Disability, and Economic Burden

Death is a rare but real outcome of paediatric micromobility trauma. National paediatric data record a case-fatality proportion of approximately 0.3% [19]. That proportion derives from weighted national estimates of emergency-department-treated injury, so its denominator comprises children who reached an emergency department and its numerator captures deaths recorded in that setting. Children who died before arrival are not captured, and the case-fatality proportion across all injured child riders is therefore likely to be underestimated. In addition, dedicated hospital series report mortality of the order of 1–2% among admitted children [4]. Longer-term morbidity is more common than death. In one hospitalised paediatric cohort, approximately one-fifth of children experienced sequelae during follow-up, and a proportion required inpatient rehabilitation [4,28]. Traumatic brain injury sustained in childhood carries the risk of persistent neurological, cognitive, and psychosocial impairment [28].

Reported costs associated with e-scooter trauma are substantial and appear to be rising, although almost none of the available evidence is paediatric. Analyses of severe e-scooter-related traumatic brain injury, predominantly in adult and mixed-age neurosurgical cohorts, demonstrate steep increases in inpatient costs over successive years, driven principally by intensive-care admission [94,95]. At the individual level, a single-centre analysis reported mean total billing charges of approximately US$95,710 per e-scooter encounter, with intoxication, intracranial haemorrhage, and traumatic brain injury independently associated with markedly higher costs [96], while a Finnish cohort estimated a total societal cost of €1.7 million, including lost productivity [97]. Three distinct quantities are conflated in this literature and should be kept apart. Billed charges are what a provider lists, hospital costs are what the care actually consumes in resources, and societal costs additionally capture lost productivity and informal care. The figure of approximately US$95,710 is a billing charge. It is not a cost, not a reimbursement, and not a measure of societal burden. Every estimate cited in this subsection derives from an adult or mixed-age cohort and therefore constitutes Category C indirect evidence in the sense of Section 2.5. Orthopaedic operative management is a particularly resource-intensive driver [98,99,100]. We could not identify a dedicated paediatric cost analysis of e-scooter or e-bike trauma, which is a substantial gap. The combination of rising numbers of presentations, frequent surgery, and intensive-care utilisation nevertheless implies a growing and under-quantified burden on paediatric trauma, surgical, and rehabilitation services [4,19].

### 6.4. Imaging and Acute Assessment

Imaging plays a central role in the acute assessment of paediatric micromobility trauma, balancing the need to identify clinically important injuries against the imperative to limit ionising radiation in children [28]. Intracranial haemorrhage is reported disproportionately often in unhelmeted children who sustain head impact, and computed-tomography-positive intracranial injury is well documented in dedicated e-scooter series [28,101]. The decision to perform cranial computed tomography should nonetheless rest on validated paediatric decision rules rather than on device type or helmet status. The rule developed by the Paediatric Emergency Care Applied Research Network identifies children at very low risk of clinically important traumatic brain injury in whom computed tomography can be avoided, and it has been compared prospectively with the CATCH and CHALICE rules [92,93]. Because cranial computed tomography in children carries a non-trivial risk of radiation-induced malignancy, we do not propose a lower imaging threshold on the basis of the injury-frequency data reviewed here. We describe the injury patterns and leave imaging thresholds to the validated rules. Cross-sectional imaging is also required to characterise orbital, maxillofacial, thoracic, and abdominal injuries when clinical findings raise concern [33,84].

A structured, multidisciplinary approach is recommended. Because craniofacial and dental injuries commonly coexist with occult brain injury, children presenting with facial trauma warrant careful neurological evaluation, with ophthalmological and maxillofacial assessment where examination findings or symptoms indicate it [76,87]. We do not propose routine speciality referral for all such children because the evidence reviewed here describes the co-occurrence of injuries and has not evaluated whether routine referral improves paediatric outcomes.

### 6.5. Rehabilitation and Long-Term Outcomes

The consequences of severe paediatric micromobility trauma frequently extend well beyond the acute admission. A meaningful minority of hospitalised children require inpatient rehabilitation, and dedicated series report that approximately one-fifth of admitted children experience sequelae during follow-up [4,28]. Among patients with e-scooter-related traumatic brain injury, a substantial proportion require protracted inpatient rehabilitation, and cognitive rehabilitation may be required after severe intracranial injury [94,95].

Because childhood traumatic brain injury is associated with long-term neurological, cognitive, behavioural, and educational sequelae, structured neuropsychological follow-up is important for affected children [28]. Nonetheless, high-quality paediatric-specific data on long-term functional and psychosocial outcomes after e-scooter and e-bike trauma remain scarce, representing a priority for future research [44]. The specific gaps are worth naming because they concern the outcomes that matter most to children and families and are the least well described. Neurocognitive recovery after e-scooter and e-bike traumatic brain injury in children has not been characterised prospectively, so the proportion of children with persisting deficits in attention, processing speed, working memory, or executive function at one and two years is unknown. Return to school, the need for educational accommodation, and academic attainment after injury are almost entirely unreported. Psychological sequelae, including post-traumatic stress symptoms, anxiety about resuming active travel, and depressive symptoms, have not been measured in this population, despite being well documented after other mechanisms of childhood injury. Health-related quality of life has not been reported using validated paediatric instruments. Outcomes after craniofacial and dental injury, which carry implications for facial growth, dentition, and appearance during adolescence, are similarly uncharacterised beyond the acute episode. Prospective paediatric cohorts with standardised outcome measurement at fixed intervals are needed to address these gaps.

## 7. E-Scooters, E-Bikes, and Conventional Devices: Comparative Injury Profiles

A recurring question in the paediatric micromobility literature is whether the electrification of scooters and bicycles independently increases injury risk and severity, over and above the injuries associated with their non-powered counterparts. The available comparative evidence, although drawn largely from single-centre and national-database studies, consistently indicates that powered devices are associated with more severe injuries [9,26,102].

### 7.1. Electric vs. Non-Electric Scooters

Direct comparisons between electric and non-electric scooters in children are consistent with a gradient of severity. In a comparative single-centre study, e-scooter riders were significantly older than users of non-electric scooters (mean 14.2 vs. 8.0 years), were significantly more likely to be involved in traffic accidents, and sustained severe head injuries more frequently, more often requiring surgical intervention [26]. Age is a major confounder in this comparison and cannot be set aside. The e-scooter group in that study was, on average, more than six years older than the non-electric scooter group, and older children ride on public roads, travel faster, are more often unsupervised, and behave differently from younger children. The reported difference in severity was not adjusted for age or for road exposure, so it cannot be attributed to electrification itself. The same limitation applies to most of the comparative literature discussed in this section, in which device type and rider age are strongly correlated by design. Analyses of national emergency-department data similarly show that injuries associated with electric scooters differ in pattern and severity from those associated with conventional scooters and bicycles [9].

These findings are consistent with the observation that children on e-scooters are more likely than those on conventional scooters to require major surgical intervention, which has been attributed to the higher speeds attainable and to the greater road exposure of powered devices, although these two explanations have not been separated in an adjusted analysis [9,26].

### 7.2. Electric vs. Conventional Bicycles

A comparable pattern is evident for bicycles. The incidence of paediatric e-bike injuries has risen over the past decade even as injuries from pedal bicycles have declined, and e-bike injuries in children tend to be more severe, more frequently requiring hospital admission or transfer [103]. In a paediatric emergency-department comparison of 196 cyclists, e-bike riders were older than manual-bicycle riders (mean 13.7 vs. 9.9 years) and sustained more intra-abdominal organ injuries, with higher-severity injuries (Injury Severity Score greater than 9) occurring exclusively among e-bike riders [102].

As with e-scooters, the capacity of e-bikes to achieve higher speeds with less physical effort than pedal bicycles is a plausible contributor to the facial fractures, intracranial haemorrhage, and intra-abdominal injury reported in children [102,103]. Helmet use is frequently invoked as a second contributor, and comparative data support the direction of the difference. In a national analysis of injuries occurring between 2017 and 2022, riders injured on electric devices were less often helmeted and more often intoxicated than riders injured on conventional devices [9]. A regional trauma-centre cohort likewise reported greater injury severity and higher hospitalisation rates after e-bike crashes than after conventional bicycle crashes [104]. Device-specific helmet-wearing rates in children remain poorly reported, however, and we could not identify a paediatric study that reports helmet prevalence separately for e-bike and conventional bicycle riders drawn from the same population. Dedicated paediatric e-bike evidence does exist, and a paediatric surgical series has characterised e-bike injury in children as an increasingly common public-health problem, but such studies remain few by comparison with the e-scooter literature [103]. Dedicated paediatric series that combine powered scooters and e-bikes report parallel increases in hospitalisations, surgical interventions, and severe trauma over time [4].

### 7.3. Speed, Motorisation, and Competing Explanations for Excess Severity

Taken together, the comparative literature is consistent with greater achievable speed being one plausible contributor to the excess severity of powered micromobility injuries in children [9,26,103]. We deliberately stop short of the stronger claim that motorisation is the principal driver, because the studies available do not support it. In every comparison summarised above, device type is confounded with rider age, and in most it is also confounded with road exposure, private ownership rather than rental, time of day, and supervision. None of the paediatric comparisons reported an analysis adjusted for these variables. A defensible summary is therefore that greater achievable speed, older rider age, greater road exposure, device geometry, and riding context are all candidate contributors, that they are correlated with one another in the available data, and that their relative contributions have not been separated. Two further asymmetries should be acknowledged. First, e-scooters and e-bikes are treated together in much of this literature and in parts of this review, yet they differ in wheel diameter, riding posture, centre of gravity, braking, legal classification, typical rider age, ownership or rental pattern, trip distance, and collision circumstance. A forward fall from a standing platform is not mechanically equivalent to a bicycle collision, and preventive measures such as rental curfews, geofencing, cycle-lane design, motor-power limits, and helmet provision apply differently to each device. Where the source studies permitted, the preceding subsections have kept the devices apart, and where they did not, the combined nature of the evidence is stated explicitly. Second, the evidence base is considerably larger for e-scooters than for e-bikes, and this review reflects that imbalance rather than correcting it. A breakdown of study counts by device type is provided in Supplementary Table S2. The practical implication for prevention is unchanged in direction but weaker in confidence than a causal reading would imply. Measures that limit speed, promote protective equipment, and restrict access by young or inexperienced riders remain reasonable across device types, and one natural experiment reports a fall in ride-based injury rates after speed and night-time restrictions were introduced [37]. Whether these measures reduce paediatric injury specifically, and by how much, has not been established [56].

## 8. Prevention and Public Health

Because paediatric e-scooter and e-bike injuries arise from a well-characterised and largely modifiable set of risk factors, many of them are, in principle, preventable [19,56]. That statement concerns potential rather than demonstrated preventability, and the evidence reviewed below distinguishes measures for which reductions in injury have actually been observed from measures proposed on mechanistic or public-health reasoning alone. Effective prevention requires a coordinated combination of legislation, protective-equipment promotion, engineering controls, safe infrastructure, and education, an approach that mirrors the successful multifaceted strategies applied to other paediatric road-traffic injuries [19,37].

### 8.1. Legislation and Regulation

Regulatory approaches to micromobility remain heterogeneous and, in many jurisdictions, incompletely enforced [56]. Commonly proposed and enacted measures include minimum-age requirements, mandatory helmet use for younger riders, restrictions on riding under the influence of alcohol, prohibition of tandem riding, and limits on where devices may be operated [56]. Age restrictions are particularly relevant to the paediatric population, given that reported injury counts are highest among younger adolescents. Several analyses explicitly support age limits as a prevention strategy [19].

Direct evidence that legislation reduces injury is accumulating. A natural-experiment study in Helsinki found that the introduction of speed and night-time usage restrictions was followed by an approximate halving of e-scooter injury rates [37], and a subsequent analysis from the same city reported that legislative restrictions were associated with a substantial reduction in e-scooter-related maxillofacial injuries [105]. These findings provide empirical support for regulatory intervention, although most such studies have been conducted in predominantly adult populations and paediatric-specific evaluations remain scarce [37,105].

### 8.2. Helmet Promotion and Protective Equipment

Given the strong association between the absence of a helmet and head and maxillofacial injury, promotion of helmet use is a central pillar of prevention [10,19]. Helmet use is associated with a lower risk of maxillofacial injury in bicycle and scooter accidents [10,106], yet helmet use among child and adolescent riders remains low, particularly with shared rental devices that are seldom supplied with helmets [56]. Expert consensus increasingly favours helmet requirements for all child riders regardless of age [19].

Practical measures to increase uptake include mandating helmet provision or availability with rental schemes, subsidised or rentable helmets, and public-education campaigns targeting both children and parents [56]. Because enforcement of helmet legislation is frequently limited, engineering and environmental measures are important complements rather than alternatives [37].

### 8.3. Speed Limitation and Engineering Controls

Speed is among the most frequently implicated correlates of injury severity, which makes speed limitation an attractive engineering target [26,37]. We avoid describing it as the most consistent determinant because the comparative studies available have not ranked candidate determinants against one another in adjusted analyses. Firmware-based speed governors on shared devices, geofenced low-speed zones, and night-time speed reductions or rental curfews directly address the periods and behaviours associated with the most severe injuries [37]. The Helsinki experience is consistent with such measures producing measurable reductions in both overall and maxillofacial injury rates, although the natural-experiment design cannot exclude concurrent changes in fleet size, seasonality, or enforcement [37,105].

Additional engineering considerations include device-design features, larger wheels, improved braking, and integrated lighting to enhance stability and visibility, together with restrictions on maximum motor power for devices marketed to or accessible by children [56].

### 8.4. Infrastructure, Education, and Behavioural Interventions

Environmental measures that separate young riders from motor traffic are likely to be especially protective, since the most severe paediatric injuries frequently involve collisions with motor vehicles [19]. Provision of protected cycle lanes and dedicated riding spaces, well-maintained road surfaces, and equitable access to safe riding infrastructure, particularly in communities with least access to safe riding space, are recurring recommendations [19]. As noted in Section 3.3, the cited evidence does not itself establish a socioeconomic gradient in injury rate, so this recommendation rests on equity and general road-safety reasoning rather than on a demonstrated disparity in risk.

Education and behavioural interventions complete the prevention framework. Targeted education of children and parents regarding device capabilities, the importance of protective equipment, and the dangers of tandem riding, alcohol use, and riding on trafficked roads is widely advocated [3,56]. A combination of educational, legislative, and environmental approaches is widely advocated [19]. It should be said plainly that no study in this field has compared education alone against a combined educational and legislative approach in a head-to-head design, so the superiority of combined approaches is inferred rather than demonstrated. What the evidence does show is that structural and legislative measures, unlike education, have been followed by measurable falls in injury. Capping the number of rental e-scooters in a Swedish city was associated with a 35% reduction in city trips and a 39% reduction in e-scooter injuries presenting to the study emergency department [62], amendments to the Road Traffic Act in the Republic of Korea have been evaluated for their effect on e-scooter injuries [63], and speed and night-time restrictions in Helsinki were followed by falls in both overall and maxillofacial injury [37,105]. We are not aware of an equivalent evaluation of an educational intervention alone in this field.

### 8.5. International Regulatory Approaches

Regulatory frameworks for e-scooters and e-bikes vary widely between and within countries, and this heterogeneity is reflected in divergent injury patterns [107]. Early regulatory responses ranged from outright prohibition, as Singapore and France banned pavement riding and the United Kingdom initially restricted e-scooters from public roads, to permissive integration with speed caps such as Belgium’s 25 km/h limit [52,108]. Minimum-age requirements are common but inconsistently enforced. In one European cohort, most injured under-age riders were below the minimum legal riding age, underscoring a critical enforcement gap [2].

Interventions that constrain exposure have measurable effects. Beyond the Helsinki speed and night-time restrictions [37,105], a Swedish study found that capping the number of rental e-scooters was followed by a 35% reduction in city trips and a 39% reduction in e-scooter injuries presenting to the study emergency department [62]. Nationwide data from Singapore likewise link device motorisation to greater injury severity and hospital admission, supporting graduated, device-specific regulation [108]. For children specifically, expert bodies increasingly advocate minimum-age rules, universal helmet requirements, and separation from motor traffic [19,56].

### 8.6. Effectiveness of Preventive Interventions

A growing body of evidence quantifies the effectiveness of preventive interventions. Helmet use has been directly associated with reduced head-injury severity in e-scooter cohorts. An Italian study demonstrated a statistically significant relationship between head-and-neck Abbreviated Injury Scale scores and trauma severity, indicating that helmet use could mitigate injury severity [109]. Legislative interventions show comparable benefit. An observational study in the Republic of Korea evaluated the effect of amendments to the Road Traffic Act on e-scooter injuries [63], while natural experiments in Finland and Sweden demonstrated reductions in injury incidence following speed, night-time, and fleet-size restrictions [37,62,105].

Comparative national studies reinforce the case for device-specific regulation. Analyses from France and Australia document the rising incidence and severity of e-scooter trauma relative to other transport modes and over time [110,111]. Collectively, these data indicate that speed limitation, fleet-size limitation, and legislative restriction have been followed by measurable reductions in injury in adult-dominated populations [10,19]. Their applicability to children is plausible but not established because none of these evaluations reported a paediatric subgroup analysis. Interpreting them for children therefore requires the Category C caveat set out in Section 2.5. These measures can be organised across the pre-event, event, and post-event phases of injury causation within a Haddon matrix framework (Table 4).

## 9. Limitations and Future Directions

The rapidly expanding literature on paediatric e-scooter and e-bike injury has generated valuable insights, but it carries important limitations that must be considered when interpreting the findings synthesised in this review.

### 9.1. Limitations of the Evidence Base

First, the evidence is dominated by retrospective single-centre series and national injury-surveillance analyses, designs susceptible to selection, referral, and coding biases that preclude reliable estimation of pooled effect sizes. This heterogeneity is precisely why formal meta-analysis has generally not been feasible [57]. Second, case definitions, age thresholds, and device classifications vary substantially between studies; some combine e-scooters and e-bikes, others merge electric and non-electric devices, and the boundary between e-bikes and higher-powered e-motos is often blurred, hampering direct comparison [4,103].

Third, much of the granular evidence on mechanisms, alcohol involvement, helmet use, and severe outcomes derives from adult or mixed-age cohorts, and its extrapolation to children is imperfect [35,95]. Fourth, exposure denominators such as rider numbers, distance travelled, or riding time are rarely available, so most studies report injury counts or emergency-department presentations rather than exposure-adjusted rates [37]. This warrants emphasis rather than a passing mention, because it is the single most consequential limitation in the field. An increase in the number of injuries does not by itself indicate an increase in the risk faced by an individual rider. If ridership has grown faster than injuries, the risk per trip may even have fallen while the reported burden rose. Shared-fleet ridership in North America grew by 31% in a single year, and private device ownership has grown in parallel, so the denominator underlying the injury counts reported in this literature has been changing rapidly and in a largely unmeasured way [14,18]. Statements about rising paediatric risk should therefore be treated as unproven, whereas statements about a rising reported burden are well supported. Fifth, surveillance systems such as the National Electronic Injury Surveillance System capture emergency-department attendances but may under-represent pre-hospital deaths and injuries managed elsewhere, and paediatric-specific data on long-term functional outcomes, rehabilitation needs, and healthcare costs remain especially sparse [19,94].

Sixth, an increase in hospital admissions cannot be read as an increase in severity without a severity denominator. Admission proportions depend on total case volume, local admission thresholds, coding practice, and bed availability as well as on injury severity, so the proportion admitted among all presentations is the more informative quantity and is reported here wherever the source studies supplied it. Seventh, many comparative statements in this literature are reported only as statistical significance, without effect estimates, confidence intervals, or the list of covariates adjusted for. Where a source study did not report these, we have said so rather than implying that an adjusted effect was demonstrated. Eighth, this review carries its own limitations. It is restricted to articles published in English, which risks omitting relevant evidence from countries with high micromobility use and substantial non-English literature. The selection of representative studies for Table 2, although governed by the explicit rules stated in Section 2.5, remains a matter of judgement and may carry emphasis bias. Finally, the field is moving quickly enough that any synthesis will date, particularly with respect to device categories such as e-motos that are increasingly accessible to children and are poorly captured by current coding systems.

### 9.2. Future Research Priorities

Priorities for future research follow directly from these gaps. Prospective, multicentre studies with standardised case definitions and age stratification are needed to characterise paediatric injury patterns and outcomes reliably [4]. Exposure-based denominators should be incorporated wherever possible to permit calculation of true incidence and to evaluate interventions [37]. Paediatric-specific evaluations of legislative and engineering measures, age limits, helmet mandates, speed governors, and infrastructure changes are required, since most existing effectiveness data derive from adult populations [37,105].

Further work should quantify the long-term neurological, functional, and psychosocial outcomes of childhood micromobility trauma, characterise the economic burden on paediatric services, and investigate the sociodemographic and structural determinants of injury to inform equitable prevention [19,94]. Finally, as device technology and regulation evolve rapidly, ongoing surveillance will be essential to track emerging injury patterns, particularly those associated with higher-powered devices increasingly accessible to children [103].

## 10. Conclusions

Electric scooters and electric bicycles are established features of childhood and adolescent mobility, and the reported burden of associated paediatric injury has increased substantially where it has been measured. Most evidence comes from retrospective clinical series and national surveillance databases, describing injury counts and emergency-department presentations rather than exposure-adjusted risk. One national analysis applying a general-population denominator found population-based injury rates rose by 293.0% for e-bikes and 88.0% for powered scooters between 2019 and 2022, so the increase is not merely an artefact of population growth. Ridership grew steeply over the same period, and whether injury probability per trip or per kilometre has changed remains unquantified in most settings.

The demographic and anatomical patterns are the most secure findings, resting on dedicated paediatric and age-stratified data. The burden falls disproportionately on adolescent boys, peaks at 11 to 14 years, and combines a predominance of extremity and soft-tissue trauma with a clinically critical minority of head, craniofacial, and severe multisystem injuries. Powered devices are associated with greater injury severity than their non-powered counterparts, but motorisation cannot be identified as the cause. In every available paediatric comparison, device type is confounded with rider age, road exposure, and riding context, and none was adjusted for these. Greater achievable speed is one plausible contributor, and prevention needs differ across device types.

A considerable part of the evidence on mechanism, alcohol involvement, cost, and intervention effectiveness is adult-derived. It supports paediatric inference without establishing it and is labelled as such throughout. Speed restriction and helmet promotion are promising, and one natural experiment reports a fall in ride-based injury rates after speed and night-time restrictions were introduced, but paediatric-specific effectiveness evidence remains limited and no intervention in this field has been evaluated in a randomised design.

A coordinated response combining age-appropriate legislation, protective-equipment provision, engineering controls, safe infrastructure, and education remains reasonable on the balance of available evidence and on general road-safety grounds. We distinguish the small group of measures for which reductions in injury have been observed, at present, essentially speed and time-of-use restriction, and the larger group proposed on mechanistic, analogical, or public-health reasoning that has not been tested in children. Prospective paediatric cohorts with exposure denominators, standardised device classification, and validated measurement of neurocognitive recovery, return to school, psychological sequelae, and quality of life are the clearest research priority. Until such cohorts exist, what is secure concerns the size and shape of the reported burden, and what is uncertain concerns why it is growing and which measures will most effectively reduce it.

## Figures and Tables

**Table 1:** Indicators of the growth in electric micromobility use and in reported injury. All values are as reported in the cited sources. Injury figures from the United States National Electronic Injury Surveillance System are weighted national estimates of emergency-department-treated injury and are not counts of all injuries.

Indicator	Reported Values	Source
Shared micromobility trips, North America	157 million (2022), 172 million (2023), at least 225 million (2024), an increase of 31% on 2023	[18]
Cities with shared micromobility systems, North America	401 (2022), 421 (2023), 415 (2024)	[18]
Shared micromobility vehicles deployed, North America	289,000 (2022), 333,000 (2024), an increase of 19% on 2023	[18]
Proportion of shared trips taken on electric devices	64% (2023), 66% (2024). E-scooters accounted for 48% of the deployed fleet in 2024	[18]
Shared e-bike trips, North America	18.8 million (2021), 30.9 million (2022)	[18]
United States e-bicycle imports	437,000 (2020), more than 1.1 million (2022)	[14]
E-scooter injuries, United States national estimate	8566 (2017), 56,847 (2022), 189,517 in total across 2017–2022	[9,15]
E-bicycle injuries, United States national estimate	751 (2017), 23,493 (2022), 45,586 in total across 2017–2022, with an estimated 5462 associated hospitalisations	[9,14,15]
Micromobility-related deaths, United States	533 across 2017–2024, rising from 5 in 2017 to 135 in 2024, comprising 206 involving e-scooters, 310 involving e-bikes, and 17 involving self-balancing scooters	[16]
Change in population-based injury rate, 2019–2022	An increase of 293.0% for e-bikes and of 88.0% for powered scooters, derived from 1,933,296 estimated injuries	[17]
Paediatric e-scooter injuries, United States national analysis	2117 across 2020–2024, of whom approximately 70% were boys and 38.3% were aged 11–14 years	[19]

**Table 3:** Pooled frequencies of injury types, severity, and mechanisms in electric scooter trauma, from systematic reviews and meta-analyses.

Injury Category/Parameter	Pooled Frequency (95% CI)	Ref.
Head and facial injuries	42.1% (38.7–45.4)	[57]
Upper-extremity injuries	40.1% (35.8–44.4)	[57]
Extremity fractures	25.7% (22.5–28.9)	[57]
Severe traumatic brain injury	2.3% (1.6–3.0)	[57]
Severe overall injury (ISS ≥ 16)	2.8% (1.5–4.1)	[57]
Mechanism: fall from device	74.4%	[88]
Mechanism: collision with a vehicle	10.2%	[88]

CI, confidence interval; ISS, Injury Severity Score. Pooled estimates are drawn from systematic reviews and meta-analyses of predominantly adult and mixed-age emergency-department cohorts.

**Table 4:** Haddon matrix of prevention strategies for paediatric electric scooter and electric bicycle injury, mapping host, vehicle, and environmental factors across the pre-event, event, and post-event phases.

Phase	Host (Child/Adolescent Rider)	Agent/Vehicle (E-Scooter or E-Bike)	Physical Environment	Socio-Economic Environment
**Pre-event (crash prevention)**	Skills training (T). Avoiding tandem riding (T). Avoiding alcohol and distraction (A).	Speed governors (A). Motor-power limits (T). Larger wheels, better brakes (T). Lighting (C).	Protected cycle lanes (C). Low-speed geofenced surfaces (A). Separation from traffic (C).	Minimum-age and licencing rules (T). Curfews (A). Fleet-size caps (T). Enforcement (C).
**Event (injury mitigation during the crash)**	Correct, well-fitted helmet use (C). Wrist guards (T).	Design limiting rider ejection and handlebar energy transfer (T).	Forgiving roadside design (C). Removal of rigid hazards (C).	Helmet mandates with helmet provision or subsidy (C).
**Post-event (outcome optimisation)**	Bystander first aid and prompt help-seeking (T).	Device crash detection and automated alerts (T).	Rapid emergency-services access (C). Trauma-system readiness (C).	Equitable access to trauma care and rehabilitation (T).

Each measure carries an evidence-status marker, since the strength of support differs greatly between them. (P) denotes evidence from an intervention study conducted in a paediatric population, (A) denotes intervention evidence from adult or mixed-age populations, (C) denotes evidence extrapolated from cycling or general road-safety research, and (T) denotes a theoretical or expert-proposed measure that has not been evaluated for injury outcomes. No measure in this matrix currently carries a (P) marker, which is itself an important finding, because it means that no prevention measure for paediatric electric micromobility injury has yet been evaluated in a dedicated paediatric intervention study. The matrix should therefore be read as a framework for organising candidate measures and research priorities rather than as a set of established recommendations.

## Data Availability

No new datasets were generated in this study. The complete database-specific search strings, a schematic of the search and selection structure, and the extraction and appraisal matrix for all included studies are provided as Supplementary Materials.

## Supplementary Materials

The following supporting information can be downloaded at: https://www.mdpi.com/article/10.3390/healthcare14152367/s1, Table S1: Complete database-specific search strategies; Table S2: Extraction and appraisal matrix for the included studies; Figure S1: Search and selection structure.

## Abbreviations

The following abbreviations are used in this manuscript.

CT	computed tomography
e-bike	electric bicycle
e-scooter	electric scooter
ED	emergency department
ISS	Injury Severity Score
NEISS	National Electronic Injury Surveillance System
PICU	paediatric intensive care unit
TBI	traumatic brain injury
