# Supplementary material for: Electric Scooter and Electric Bicycle Injuries in Children and Adolescents: A Narrative Review of Epidemiology, Injury Patterns, Clinical Outcomes, and Prevention
Source: Healthcare (Basel). 2026 Aug 3;14(15):2367. doi: 10.3390/healthcare14152367 (PMC13463875; doi:10.3390/healthcare14152367)
Supplement: Supplementary file 1 [file healthcare-14-02367-s001.zip › healthcare-4456224-supplementary.pdf]

Supplementary Materials

Electric Scooter and Electric Bicycle Injuries in Children and Adolescents. A Narrative Review of Epidemiology, Injury Patterns, Clinical Outcomes, and Prevention

Table S1. Complete database-specific search strategies.

Block structure. Block 1 (device) AND Block 2 (outcome) AND Block 3 (population). Terms within each block are combined with OR. All four databases were last searched on 30 June 2026.

Database	Search strategy	Limits and coverage
PubMed/MEDLINE	Block 1: ("electric scooter"[tiab] OR "electric scooters"[tiab] OR "e-scooter"[tiab] OR "e-scooters"[tiab] OR "powered scooter"[tiab] OR "standing scooter"[tiab] OR "motorized scooter"[tiab] OR "motorised scooter"[tiab] OR "electric bicycle"[tiab] OR "electric bicycles"[tiab] OR "e-bike"[tiab] OR "e-bikes"[tiab] OR "pedelec"[tiab] OR "pedelecs"[tiab] OR "micromobility"[tiab] OR "micro-mobility"[tiab])<br>Block 2: (injur*[tiab] OR trauma*[tiab] OR fracture*[tiab] OR concussion*[tiab] OR accident*[tiab] OR crash*[tiab] OR wound*[tiab] OR "wounds and injuries"[mh])<br>Block 3: (pediatric*[tiab] OR paediatric*[tiab] OR child*[tiab] OR children[tiab] OR adolescen*[tiab] OR teen*[tiab] OR youth[tiab] OR "school age"[tiab] OR child[mh] OR adolescent[mh] OR infant[mh])	Limits: english[lang]. Coverage: database inception to 30 June 2026.
Scopus	TITLE-ABS-KEY(("electric scooter" OR "electric scooters" OR "e-scooter" OR "e-scooters" OR "powered scooter" OR "standing scooter" OR "motorized scooter" OR "motorised scooter" OR "electric bicycle" OR "electric bicycles" OR "e-bike" OR "e-bikes" OR pedelec OR pedelecs OR micromobility OR "micro-mobility")) AND TITLE-ABS-KEY((injur* OR trauma* OR fracture* OR concussion* OR accident* OR crash* OR wound*)) AND TITLE-ABS-KEY((pediatric* OR paediatric* OR child* OR children OR adolescen* OR teen* OR youth OR "school age")) AND (LIMIT-TO(LANGUAGE,"English"))	Coverage: database inception to 30 June 2026.
Web of Science Core Collection	TS=("electric scooter" OR "electric scooters" OR "e-scooter" OR "e-scooters" OR "powered scooter" OR "standing scooter" OR "motorized scooter" OR "motorised scooter" OR "electric bicycle" OR "electric bicycles" OR "e-bike" OR "e-bikes" OR pedelec OR pedelecs OR micromobility OR "micro-mobility") AND TS=(injur* OR trauma* OR fracture* OR concussion* OR accident* OR crash* OR wound*) AND TS=(pediatric* OR paediatric* OR child* OR children OR adolescen* OR teen* OR youth OR "school age")	Language: English. Coverage: database inception to 30 June 2026.

Google Scholar	("electric scooter" OR "e-scooter" OR "electric bicycle" OR "e-bike" OR micromobility) AND (injury OR trauma OR fracture) AND (pediatric OR paediatric OR child OR adolescent)	Screening was restricted to the first 200 results ranked by relevance. Date searched: 30 June 2026.

Parallel search omitting Block 3. The Block 1 AND Block 2 search was also run in each database without the population block, in order to capture mixed-age studies carrying no paediatric indexing terms and studies used as indirect contextual evidence.

Hand-searching and citation tracking. Reference lists of all included articles and of relevant reviews were screened, and forward citation tracking was performed on the most frequently cited articles.

Figure S1. Search and selection structure.

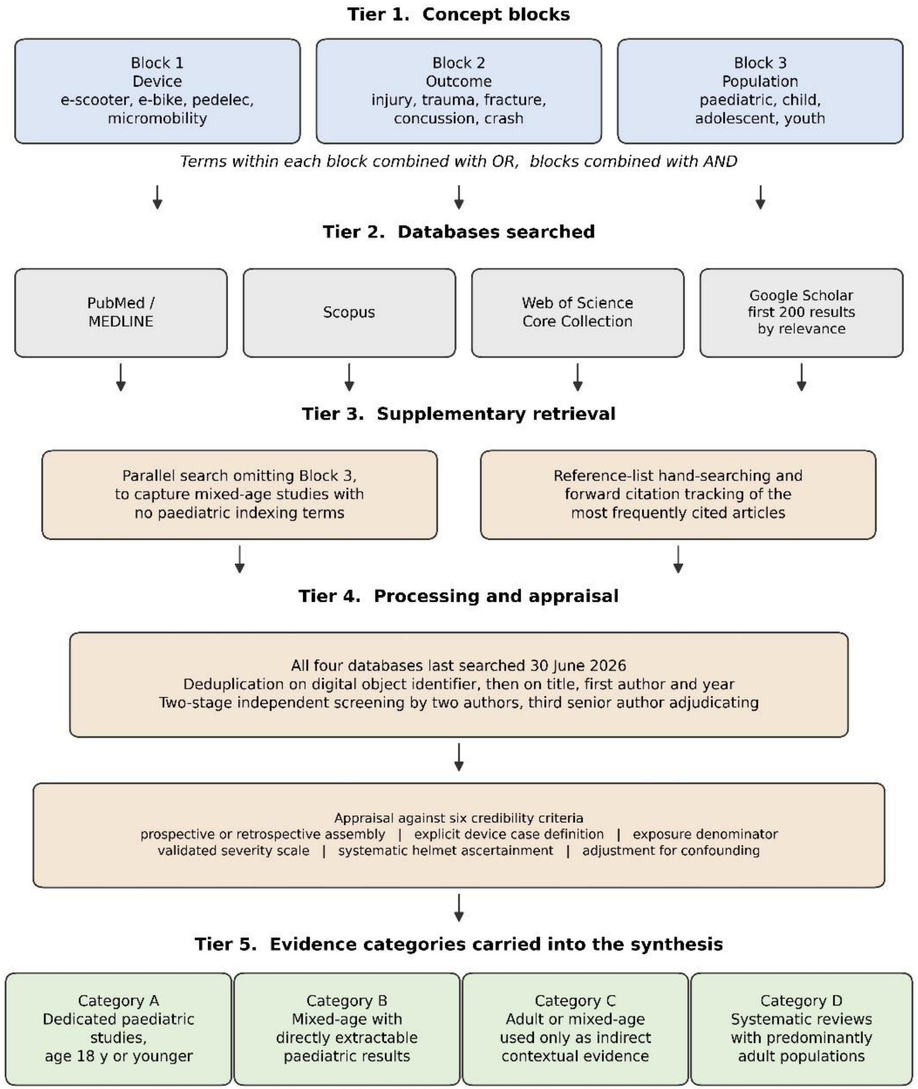

A conceptual schematic of the search and selection process, read in five tiers from top to bottom. Tier 1 shows the three concept blocks with their terms. Tier 2 shows the Boolean combination rule. Tier 3 shows the four databases searched. Tier 4 shows the parallel search omitting the population block, the hand-searching and forward citation tracking, the common search date of 30 June 2026, deduplication, two-stage independent screening by two authors, and appraisal against the six credibility criteria. Tier 5 shows the four evidence categories as outputs, namely Category A dedicated paediatric studies, Category B mixed-age studies with extractable paediatric results, Category C adult or mixed-age studies used as indirect contextual evidence, and Category D adult-dominated systematic reviews.

Table S2. Extraction and appraisal matrix for the included studies.

Scope and design. This matrix covers the 105 cited sources that report primary or synthesised evidence. Six cited items are excluded because they are not studies, namely the SANRA appraisal instrument, a published correction notice, a two-page commentary, reports from the United States Consumer Product Safety Commission and from the North American Bikeshare and Scootershare Association, and a 2000 news item. For each source the matrix records the device type as reported by the source, the manuscript sections in which that source is principally used, the evidence category defined in Section 2.5 of the manuscript, and the interpretative weight assigned to it in the synthesis together with the reason for that weight. Rows are ordered by reference number so that every entry can be checked directly against the reference list of the manuscript. Where a cell reads not device-specific, or states that the powered status was not specified in the source, that is the reported position of the source rather than a missing entry.

Study (first author, year)	Ref.	Device type	Manuscript sections of principal use	Evidence category	Interpretative weight and reason
Namiri et al. (2020)	[1]	E-scooter	1. Introduction; 3.1. International Trends in Reported Injuries and Emergency Presentations; 3.2. Age and Sex Distribution	C	Lower. Mixed-age or adult study used as indirect context
Coelho et al. (2021)	[2]	E-scooter	1. Introduction; 3.6. The COVID-19 Pandemic and Micromobility Injury; 8.5. International Regulatory Approaches	C	Lower. Mixed-age or adult study used as indirect context
Moati et al. (2025)	[4]	E-scooter and e-bike	1. Introduction; 3.1. International Trends in Reported Injuries and Emergency Presentations; 3.2. Age and Sex Distribution; 3.4. Early and Frequently Cited Studies and International Cohorts; 5. Injury Patterns and Anatomical Distribution; 6. Clinical Management and Outcomes; 6.2. Hospitalisation, Surgery, and Intensive Care; 6.3. Mortality, Disability, and Economic Burden; 6.5.	A	Higher. Dedicated paediatric study

			Rehabilitation and Long-Term Outcomes; 7.2. Electric versus Conventional Bicycles; 9.1. Limitations of the Evidence Base; 9.2. Future Research Priorities		
Weiss et al. (2026)	[5]	E-scooter	1. Introduction; 3.1. International Trends in Reported Injuries and Emergency Presentations; 3.6. The COVID-19 Pandemic and Micromobility Injury	A	Higher. Dedicated paediatric study
Schuller et al. (2023)	[6]	Non-powered scooter	1. Introduction; 3.2. Age and Sex Distribution; 5. Injury Patterns and Anatomical Distribution; 5.1. Overall Anatomical Distribution; 5.5. Truncal, Abdominal, and Spinal Injuries	A	Higher. Dedicated paediatric study
Reito et al. (2022)	[7]	E-scooter	1. Introduction; 3.1. International Trends in Reported Injuries and Emergency Presentations	C	Lower. Mixed-age or adult study used as indirect context
Shichman et al. (2022)	[8]	E-scooter	1. Introduction; 3.1. International Trends in Reported Injuries and Emergency Presentations; 3.6. The COVID-19 Pandemic and Micromobility Injury	C	Lower. Mixed-age or adult study used as indirect context
Fernandez et al. (2024)	[9]	E-scooter	1. Introduction; 3.1. International Trends in Reported Injuries and Emergency Presentations; 7. E-scooters, E-bikes, and Conventional Devices: Comparative Injury Profiles; 7.1. Electric versus Non-electric Scooters; 7.2. Electric versus Conventional Bicycles; 7.3. Speed,	C	Lower. Mixed-age or adult study used as indirect context

			Motorisation, and Competing Explanations for Excess Severity		
Stassen et al. (2024)	[10]	Bicycle	1. Introduction; 4.2. Helmet Use and Protective Equipment; 5.3. Craniofacial and Dental Injuries; 8.2. Helmet Promotion and Protective Equipment; 8.6. Effectiveness of Preventive Interventions	D	Lower. Adult-dominated synthesis, indirect evidence
Goh et al. (2023)	[11]	E-scooter	1. Introduction; 5.3. Craniofacial and Dental Injuries; 6.1. Emergency Presentation and Acute Management; 6.2. Hospitalisation, Surgery, and Intensive Care	D	Lower. Adult-dominated synthesis, indirect evidence
Dhillon et al. (2020)	[12]	E-scooter	1. Introduction	C	Lower. Mixed-age or adult study used as indirect context
Fernandez et al. (2024)	[14]	E-bike	3.1. International Trends in Reported Injuries and Emergency Presentations; 9.1. Limitations of the Evidence Base	C	Lower. Mixed-age or adult study used as indirect context
Burford et al. (2024)	[17]	E-scooter and e-bike	3.1. International Trends in Reported Injuries and Emergency Presentations	C	Lower. Mixed-age or adult study used as indirect context
Howard et al. (2026)	[19]	E-scooter	3.1. International Trends in Reported Injuries and Emergency Presentations; 3.2. Age and Sex Distribution; 3.3. Sociodemographic Disparities; 4. Mechanisms and Risk Factors; 4.2. Helmet Use and Protective Equipment; 4.3. Speed, Riding Behaviour, and	A	Higher. Dedicated paediatric study

			Environmental Factors; 4.5. Behavioural and Human Factors; 5. Injury Patterns and Anatomical Distribution; 5.1. Overall Anatomical Distribution; 6. Clinical Management and Outcomes; 6.1. Emergency Presentation and Acute Management; 6.2. Hospitalisation, Surgery, and Intensive Care; 6.3. Mortality, Disability, and Economic Burden; 8. Prevention and Public Health; 8.1. Legislation and Regulation; 8.2. Helmet Promotion and Protective Equipment; 8.4. Infrastructure, Education, and Behavioural Interventions; 8.5. International Regulatory Approaches; 8.6. Effectiveness of Preventive Interventions; 9.1. Limitations of the Evidence Base; 9.2. Future Research Priorities		
Levine et al. (2001)	[20]	Non-powered scooter	3.1. International Trends in Reported Injuries and Emergency Presentations	A	Lower. Non-powered scooter, used only for the historical baseline
Schalamon et al. (2003)	[21]	Non-powered scooter	3.1. International Trends in Reported Injuries and Emergency Presentations	A	Lower. Non-powered scooter, used only for the historical baseline
Fong et al. (2004)	[22]	Non-powered scooter	3.1. International Trends in Reported Injuries and Emergency Presentations	A	Lower. Non-powered scooter, used only for the historical baseline
Anderson-Suddarth et al. (2005)	[23]	Non-powered scooter	3.1. International Trends in Reported Injuries and Emergency Presentations	A	Lower. Non-powered scooter, used only for the historical baseline

Mebert et al. (2018)	[25]	Non-powered scooter	3.1. International Trends in Reported Injuries and Emergency Presentations	C	Lower. Non-powered scooter, used only for the historical baseline
Schuller et al. (2025)	[26]	E-scooter	3.1. International Trends in Reported Injuries and Emergency Presentations; 3.2. Age and Sex Distribution; 4.3. Speed, Riding Behaviour, and Environmental Factors; 5.2. Traumatic Brain Injury and Head Trauma; 7. E-scooters, E-bikes, and Conventional Devices: Comparative Injury Profiles; 7.1. Electric versus Non-electric Scooters; 7.3. Speed, Motorisation, and Competing Explanations for Excess Severity; 8.3. Speed Limitation and Engineering Controls	A	Higher. Dedicated paediatric study
Cohen et al. (2022)	[27]	E-scooter	3.1. International Trends in Reported Injuries and Emergency Presentations; 3.6. The COVID-19 Pandemic and Micromobility Injury	A	Higher. Dedicated paediatric study
Abu-Kishk et al. (2025)	[28]	E-scooter	3.2. Age and Sex Distribution; 4.2. Helmet Use and Protective Equipment; 5. Injury Patterns and Anatomical Distribution; 5.1. Overall Anatomical Distribution; 5.2. Traumatic Brain Injury and Head Trauma; 5.4. Extremity and Orthopaedic Injuries; 5.5. Truncal, Abdominal, and Spinal Injuries; 6. Clinical Management and Outcomes; 6.1. Emergency Presentation and Acute	A	Higher. Dedicated paediatric study

			Management; 6.2. Hospitalisation, Surgery, and Intensive Care; 6.3. Mortality, Disability, and Economic Burden; 6.4. Imaging and Acute Assessment; 6.5. Rehabilitation and Long-Term Outcomes		
Trivedi et al. (2019)	[29]	E-scooter	3.2. Age and Sex Distribution; 3.4. Early and Frequently Cited Studies and International Cohorts	C	Lower. Mixed-age or adult study used as indirect context
Weiss et al. (2025)	[30]	E-scooter	3.2. Age and Sex Distribution; 5.4. Extremity and Orthopaedic Injuries	A	Higher. Dedicated paediatric study
Büyükceran et al. (2023)	[31]	E-scooter	3.2. Age and Sex Distribution; 5.4. Extremity and Orthopaedic Injuries	B	Higher. Mixed-age study with extractable paediatric results
Kleinertz et al. (2023)	[32]	E-scooter	3.2. Age and Sex Distribution; 3.4. Early and Frequently Cited Studies and International Cohorts; 3.5. Non-Rider and Bystander Injuries; 4.1. Mechanisms of Injury; 4.2. Helmet Use and Protective Equipment; 4.4. Alcohol and Substance Use	C	Lower. Mixed-age or adult study used as indirect context
Cirillo et al. (2025)	[33]	E-scooter	3.2. Age and Sex Distribution; 3.4. Early and Frequently Cited Studies and International Cohorts; 6.4. Imaging and Acute Assessment	B	Higher. Mixed-age study with extractable paediatric results
Foglar et al. (2025)	[34]	E-scooter	3.2. Age and Sex Distribution; 3.4. Early and Frequently Cited Studies and International Cohorts	C	Lower. Mixed-age or adult study used as indirect context

Suominen et al. (2022)	[35]	E-scooter	3.2. Age and Sex Distribution; 4.3. Speed, Riding Behaviour, and Environmental Factors; 4.4. Alcohol and Substance Use; 5.2. Traumatic Brain Injury and Head Trauma; 9.1. Limitations of the Evidence Base	C	Lower. Mixed-age or adult study used as indirect context
Ruan et al. (2024)	[36]	E-scooter	3.2. Age and Sex Distribution; 5.6. Ocular and Periocular Injuries	C	Lower. Mixed-age or adult study used as indirect context
Pakarinen et al. (2023)	[37]	E-scooter	3.2. Age and Sex Distribution; 4.3. Speed, Riding Behaviour, and Environmental Factors; 7.3. Speed, Motorisation, and Competing Explanations for Excess Severity; 8. Prevention and Public Health; 8.1. Legislation and Regulation; 8.2. Helmet Promotion and Protective Equipment; 8.3. Speed Limitation and Engineering Controls; 8.4. Infrastructure, Education, and Behavioural Interventions; 8.5. International Regulatory Approaches; 8.6. Effectiveness of Preventive Interventions; 9.1. Limitations of the Evidence Base; 9.2. Future Research Priorities	C	Lower. Mixed-age or adult study used as indirect context
Kobayashi et al. (2019)	[38]	E-scooter	3.4. Early and Frequently Cited Studies and International Cohorts	C	Lower. Mixed-age or adult study used as indirect context
Brownson et al. (2019)	[39]	E-scooter	3.4. Early and Frequently Cited Studies and International Cohorts	C	Lower. Mixed-age or adult study used as indirect context

Suslavičius et al. (2024)	[40]	E-scooter	3.4. Early and Frequently Cited Studies and International Cohorts	C	Lower. Mixed-age or adult study used as indirect context
Begum et al. (2025)	[41]	E-scooter	3.4. Early and Frequently Cited Studies and International Cohorts	C	Lower. Mixed-age or adult study used as indirect context
Metry et al. (2024)	[42]	E-scooter	3.4. Early and Frequently Cited Studies and International Cohorts	C	Lower. Mixed-age or adult study used as indirect context
Seitakari et al. (2025)	[43]	E-scooter	3.4. Early and Frequently Cited Studies and International Cohorts	C	Lower. Mixed-age or adult study used as indirect context
Cho et al. (2024)	[44]	Scooter, powered status not specified in source title	3.4. Early and Frequently Cited Studies and International Cohorts; 6.5. Rehabilitation and Long-Term Outcomes	C	Lower. Mixed-age or adult study used as indirect context
Puzio et al. (2020)	[45]	E-scooter	3.4. Early and Frequently Cited Studies and International Cohorts	C	Lower. Mixed-age or adult study used as indirect context
Sikka et al. (2019)	[46]	E-scooter	3.5. Non-Rider and Bystander Injuries	C	Lower. Mixed-age or adult study used as indirect context
Siman-Tov et al. (2017)	[47]	E-bike	3.5. Non-Rider and Bystander Injuries	C	Lower. Mixed-age or adult study used as indirect context
Jafari et al. (2024)	[48]	E-scooter	3.5. Non-Rider and Bystander Injuries	C	Lower. Background source outside the micromobility injury field
Blomberg et al. (2019)	[49]	E-scooter	3.6. The COVID-19 Pandemic and Micromobility Injury	C	Lower. Mixed-age or adult study used as indirect context
Badeau et al. (2019)	[50]	E-scooter	3.6. The COVID-19 Pandemic and Micromobility Injury	C	Lower. Mixed-age or adult study used as indirect context
Northway et al. (2022)	[51]	E-scooter	3.6. The COVID-19 Pandemic and Micromobility Injury	C	Lower. Mixed-age or adult study used as indirect context

Gan-El et al. (2022)	[52]	E-scooter	3.6. The COVID-19 Pandemic and Micromobility Injury; 8.5. International Regulatory Approaches	C	Lower. Mixed-age or adult study used as indirect context
Leyendecker et al. (2023)	[53]	E-scooter	3.6. The COVID-19 Pandemic and Micromobility Injury	C	Lower. Mixed-age or adult study used as indirect context
Hazra et al. (2021)	[54]	Not device-specific	3.6. The COVID-19 Pandemic and Micromobility Injury	C	Lower. Mixed-age or adult study used as indirect context
Navon et al. (2023)	[55]	Bicycle	3.6. The COVID-19 Pandemic and Micromobility Injury	C	Lower. Mixed-age or adult study used as indirect context
Nemeh et al. (2025)	[56]	E-scooter	4. Mechanisms and Risk Factors; 4.1. Mechanisms of Injury; 4.2. Helmet Use and Protective Equipment; 4.4. Alcohol and Substance Use; 7.3. Speed, Motorisation, and Competing Explanations for Excess Severity; 8. Prevention and Public Health; 8.1. Legislation and Regulation; 8.2. Helmet Promotion and Protective Equipment; 8.3. Speed Limitation and Engineering Controls; 8.4. Infrastructure, Education, and Behavioural Interventions; 8.5. International Regulatory Approaches	C	Lower. Mixed-age or adult study used as indirect context
Käckenmester et al. (2026)	[57]	E-scooter	4.1. Mechanisms of Injury; 4.2. Helmet Use and Protective Equipment; 4.3. Speed, Riding Behaviour, and Environmental Factors; 4.4. Alcohol and Substance Use; 5.7. Evidence from Systematic Reviews and	D	Lower. Adult-dominated synthesis, indirect evidence

			Meta-Analyses; 9.1. Limitations of the Evidence Base		
Uluk et al. (2022)	[58]	E-scooter	4.1. Mechanisms of Injury	C	Lower. Mixed-age or adult study used as indirect context
Neuroth et al. (2022)	[59]	E-scooter	4.1. Mechanisms of Injury; 5.5. Truncal, Abdominal, and Spinal Injuries	C	Lower. Mixed-age or adult study used as indirect context
Gudeman et al. (2022)	[60]	Scooter, powered status not specified in source title	4.3. Speed, Riding Behaviour, and Environmental Factors	C	Lower. Mixed-age or adult study used as indirect context
Burt et al. (2023)	[61]	E-scooter	4.5. Behavioural and Human Factors	C	Lower. Mixed-age or adult study used as indirect context
Liu et al. (2025)	[62]	E-scooter	4.5. Behavioural and Human Factors; 8.4. Infrastructure, Education, and Behavioural Interventions; 8.5. International Regulatory Approaches; 8.6. Effectiveness of Preventive Interventions	C	Lower. Mixed-age or adult study used as indirect context
Jeong et al. (2022)	[63]	E-scooter	4.5. Behavioural and Human Factors; 8.4. Infrastructure, Education, and Behavioural Interventions; 8.6. Effectiveness of Preventive Interventions	C	Lower. Mixed-age or adult study used as indirect context
Mahdavi Sharif et al. (2023)	[64]	Bicycle	4.5. Behavioural and Human Factors	D	Lower. Background source outside the micromobility injury field
Osti et al. (2023)	[65]	E-scooter and e-bike	5.1. Overall Anatomical Distribution	C	Lower. Mixed-age or adult study used as indirect context
Fisher et al. (2023)	[66]	E-scooter	5.1. Overall Anatomical Distribution	C	Lower. Mixed-age or adult study used as indirect context

Akalin et al. (2023)	[67]	E-scooter	5.3. Craniofacial and Dental Injuries	C	Lower. Mixed-age or adult study used as indirect context
Kowalczewska et al. (2023)	[68]	E-scooter	5.3. Craniofacial and Dental Injuries	C	Lower. Mixed-age or adult study used as indirect context
Grill et al. (2022)	[69]	E-scooter	5.3. Craniofacial and Dental Injuries	C	Lower. Mixed-age or adult study used as indirect context
Piccolino et al. (2024)	[70]	E-scooter	5.3. Craniofacial and Dental Injuries	C	Lower. Mixed-age or adult study used as indirect context
Sritharan et al. (2023)	[71]	E-scooter	5.3. Craniofacial and Dental Injuries	C	Lower. Mixed-age or adult study used as indirect context
Arbel et al. (2022)	[72]	E-scooter	5.3. Craniofacial and Dental Injuries	C	Lower. Mixed-age or adult study used as indirect context
Dhar et al. (2024)	[73]	E-scooter	5.3. Craniofacial and Dental Injuries	C	Lower. Mixed-age or adult study used as indirect context
Ahluwalia et al. (2023)	[74]	E-scooter	5.3. Craniofacial and Dental Injuries	C	Lower. Mixed-age or adult study used as indirect context
Kwon et al. (2024)	[75]	E-scooter	5.3. Craniofacial and Dental Injuries	C	Lower. Mixed-age or adult study used as indirect context
Kim et al. (2021)	[76]	E-scooter	5.3. Craniofacial and Dental Injuries; 6.1. Emergency Presentation and Acute Management; 6.4. Imaging and Acute Assessment	C	Lower. Mixed-age or adult study used as indirect context
Siow et al. (2020)	[77]	E-scooter	5.4. Extremity and Orthopaedic Injuries	C	Lower. Mixed-age or adult study used as indirect context
Morgan et al. (2022)	[78]	E-scooter	5.4. Extremity and Orthopaedic Injuries	A	Higher. Dedicated paediatric study
Azab et al. (2022)	[79]	E-scooter	5.5. Truncal, Abdominal, and Spinal Injuries	D	Lower. Adult-dominated synthesis, indirect evidence

Rashed et al. (2022)	[80]	E-scooter	5.5. Truncal, Abdominal, and Spinal Injuries	C	Lower. Mixed-age or adult study used as indirect context
Schlaff et al. (2019)	[81]	E-scooter	5.5. Truncal, Abdominal, and Spinal Injuries	C	Lower. Mixed-age or adult study used as indirect context
Lin et al. (2023)	[82]	E-scooter	5.5. Truncal, Abdominal, and Spinal Injuries	C	Lower. Mixed-age or adult study used as indirect context
Özlü et al. (2025)	[83]	E-scooter	5.5. Truncal, Abdominal, and Spinal Injuries	C	Lower. Mixed-age or adult study used as indirect context
Yarmohammadi et al. (2020)	[84]	E-scooter	5.6. Ocular and Periocular Injuries; 6.4. Imaging and Acute Assessment	C	Lower. Mixed-age or adult study used as indirect context
Haring et al. (2016)	[85]	Not device-specific	5.6. Ocular and Periocular Injuries	C	Lower. Background source outside the micromobility injury field
McLaughlin et al. (2025)	[86]	E-scooter	5.6. Ocular and Periocular Injuries	C	Lower. Mixed-age or adult study used as indirect context
Haque et al. (2026)	[87]	E-scooter	5.6. Ocular and Periocular Injuries; 6.4. Imaging and Acute Assessment	C	Lower. Mixed-age or adult study used as indirect context
Singh et al. (2022)	[88]	E-scooter	5.7. Evidence from Systematic Reviews and Meta-Analyses	D	Lower. Adult-dominated synthesis, indirect evidence
Spota et al. (2024)	[89]	E-scooter	5.7. Evidence from Systematic Reviews and Meta-Analyses	D	Lower. Adult-dominated synthesis, indirect evidence
Niemann et al. (2023)	[90]	Combined micromobility	5.7. Evidence from Systematic Reviews and Meta-Analyses	D	Lower. Adult-dominated synthesis, indirect evidence
Iroz-Elardo et al. (2021)	[91]	E-scooter	5.7. Evidence from Systematic Reviews and Meta-Analyses	C	Lower. Mixed-age or adult study used as indirect context
Kuppermann et al. (2009)	[92]	Not device-specific	6.1. Emergency Presentation and Acute Management; 6.4. Imaging and Acute Assessment	A	Lower. Background source outside the micromobility injury field

Easter et al. (2014)	[93]	Not device-specific	6.1. Emergency Presentation and Acute Management; 6.4. Imaging and Acute Assessment	A	Lower. Background source outside the micromobility injury field
Chang et al. (2026)	[94]	E-scooter	6.2. Hospitalisation, Surgery, and Intensive Care; 6.3. Mortality, Disability, and Economic Burden; 6.5. Rehabilitation and Long-Term Outcomes; 9.1. Limitations of the Evidence Base; 9.2. Future Research Priorities	C	Lower. Mixed-age or adult study used as indirect context
McKay et al. (2023)	[95]	E-scooter	6.3. Mortality, Disability, and Economic Burden; 6.5. Rehabilitation and Long-Term Outcomes; 9.1. Limitations of the Evidence Base	C	Lower. Mixed-age or adult study used as indirect context
Lavoie-Gagne et al. (2021)	[96]	E-scooter	6.3. Mortality, Disability, and Economic Burden	C	Lower. Mixed-age or adult study used as indirect context
Vasara et al. (2022)	[97]	E-scooter	6.3. Mortality, Disability, and Economic Burden	C	Lower. Mixed-age or adult study used as indirect context
English et al. (2020)	[98]	E-scooter	6.3. Mortality, Disability, and Economic Burden	C	Lower. Mixed-age or adult study used as indirect context
Lavoie-Gagne et al. (2021)	[99]	E-scooter	6.3. Mortality, Disability, and Economic Burden	C	Lower. Mixed-age or adult study used as indirect context
Antonik et al. (2024)	[100]	E-scooter	6.3. Mortality, Disability, and Economic Burden	C	Lower. Mixed-age or adult study used as indirect context
Bareikis et al. (2024)	[101]	E-scooter	6.4. Imaging and Acute Assessment	C	Lower. Mixed-age or adult study used as indirect context
Capua et al. (2019)	[102]	E-bike	7. E-scooters, E-bikes, and Conventional Devices: Comparative Injury Profiles;	A	Higher. Dedicated paediatric study

			7.2. Electric versus Conventional Bicycles		
Goodman et al. (2023)	[103]	E-bike	7.2. Electric versus Conventional Bicycles; 7.3. Speed, Motorisation, and Competing Explanations for Excess Severity; 9.1. Limitations of the Evidence Base; 9.2. Future Research Priorities	A	Higher. Dedicated paediatric study
Berk et al. (2022)	[104]	E-bike	7.2. Electric versus Conventional Bicycles	C	Lower. Mixed-age or adult study used as indirect context
Snäll et al. (2025)	[105]	E-scooter	8.1. Legislation and Regulation; 8.3. Speed Limitation and Engineering Controls; 8.4. Infrastructure, Education, and Behavioural Interventions; 8.5. International Regulatory Approaches; 8.6. Effectiveness of Preventive Interventions; 9.2. Future Research Priorities	C	Lower. Mixed-age or adult study used as indirect context
Abu-Kishk et al. (2010)	[106]	Bicycle	8.2. Helmet Promotion and Protective Equipment	A	Lower. Background source outside the micromobility injury field
Kłosiewicz et al. (2024)	[107]	E-scooter	8.5. International Regulatory Approaches	C	Lower. Mixed-age or adult study used as indirect context
Tan et al. (2019)	[108]	Combined micromobility	8.5. International Regulatory Approaches	C	Lower. Mixed-age or adult study used as indirect context
Cittadini et al. (2023)	[109]	E-scooter	8.6. Effectiveness of Preventive Interventions	C	Lower. Mixed-age or adult study used as indirect context
Jamieson et al. (2023)	[110]	E-scooter	8.6. Effectiveness of Preventive Interventions	C	Lower. Mixed-age or adult study used as indirect context

James et al. (2023)	[111]	E-scooter	8.6. Effectiveness of Preventive Interventions	C	Lower. Mixed-age or adult study used as indirect context

Counts by device type. E-scooter 79, e-bike 5, e-scooter and e-bike 3, non-powered scooter 6, bicycle 4, combined micromobility 2, scooter with powered status not specified in the source 2, and not device-specific 4. **Counts by evidence category.** Category A 18, Category B 2, Category C 77, Category D 8.
